# Stromal remodeling by the BET bromodomain inhibitor JQ1 suppresses the progression of human pancreatic cancer

**DOI:** 10.18632/oncotarget.11129

**Published:** 2016-08-09

**Authors:** Keisuke Yamamoto, Keisuke Tateishi, Yotaro Kudo, Mayumi Hoshikawa, Mariko Tanaka, Takuma Nakatsuka, Hiroaki Fujiwara, Koji Miyabayashi, Ryota Takahashi, Yasuo Tanaka, Hideaki Ijichi, Yousuke Nakai, Hiroyuki Isayama, Yasuyuki Morishita, Taku Aoki, Yoshihiro Sakamoto, Kiyoshi Hasegawa, Norihiro Kokudo, Masashi Fukayama, Kazuhiko Koike

**Affiliations:** ^1^ Department of Gastroenterology, Graduate School of Medicine, The University of Tokyo, Bunkyo-ku, Tokyo 113-8655, Japan; ^2^ Hepato-Biliary-Pancreatic Surgery Division, Department of Surgery, Graduate School of Medicine, The University of Tokyo, Bunkyo-ku, Tokyo 113-8655, Japan; ^3^ Department of Pathology and Diagnostic Pathology, Graduate School of Medicine, The University of Tokyo, Bunkyo-ku, Tokyo 113-8655, Japan; ^4^ Department of Molecular Pathology, Graduate School of Medicine, The University of Tokyo, Bunkyo-ku, Tokyo 113-8655, Japan; ^5^ Second Department of Surgery, Dokkyo Medical University, Mibu, Tochigi 321-0293, Japan

**Keywords:** pancreatic ductal adenocarcinoma (PDAC), cancer-associated fibroblast (CAF), epigenetics, bromodomain and extraterminal domain (BET) proteins, JQ1

## Abstract

Inhibitors of bromodomain and extraterminal domain (BET) proteins, a family of chromatin reader proteins, have therapeutic efficacy against various malignancies. However, the detailed mechanisms underlying the anti-tumor effects in distinct tumor types remain elusive. Here, we show a novel antitumor mechanism of BET inhibition in pancreatic ductal adenocarcinoma (PDAC). We found that JQ1, a BET inhibitor, decreased desmoplastic stroma, a hallmark of PDAC, and suppressed the growth of patient-derived tumor xenografts (PDX) of PDACs. *In vivo* antitumor effects of JQ1 were not always associated with the JQ1 sensitivity of respective PDAC cells, and were rather dependent on the suppression of tumor-promoting activity in cancer-associated fibroblasts (CAFs). JQ1 inhibited Hedgehog and TGF-β pathways as potent regulators of CAF activation and suppressed the expression of α-SMA, extracellular matrix, cytokines, and growth factors in human primary CAFs. Consistently, conditioned media (CM) from CAFs promoted the proliferation of PDAC cells along with the activation of ERK, AKT, and STAT3 pathways, though these effects were suppressed when CM from JQ1-treated CAFs was used. Mechanistically, chromatin immunoprecipitation experiments revealed that JQ1 reduced TGF-β–dependent gene expression by disrupting the recruitment of the transcriptional machinery containing BET proteins. Finally, combination therapy with gemcitabine plus JQ1 showed greater efficacy than gemcitabine monotherapy against PDAC *in vivo*. Thus, our results reveal BET proteins as the critical regulators of CAF-activation and also provide evidence that stromal remodeling by epigenetic modulators can be a novel therapeutic option for PDAC.

## INTRODUCTION

Pancreatic ductal adenocarcinoma (PDAC) is a deadly disease characterized by abundant desmoplastic stroma [1]. Cancer-associated fibroblasts (CAFs) are the most abundant cell types in the tumor stroma. During tumorigenesis, CAFs are activated by soluble factors like hedgehog (Hh) ligands or TGF-β, which are secreted from PDAC cells [1]. Activated CAFs acquire tumor-promoting properties such as enhanced extracellular matrix (ECM) synthesis and increased secretion of growth factors and inflammatory cytokines [2]. As CAFs have been implicated in disease progression and therapeutic resistance, CAFs have long attracted attention as a therapeutic target in PDAC. However, the precise mechanisms by which CAFs are activated and maintain activated phenotypes remain elusive.

The bromodomain and extraterminal domain (BET) family proteins, BRD2, BRD3, BRD4, and BRDT, recognize acetylated lysine residues on histone tails and recruit transcriptional regulatory complexes, facilitating gene transcription by RNA polymerase II (Pol II) [2]. Recently, selective small molecule inhibitors of BET proteins, such as JQ1 [3] and I-BETs [4], have demonstrated remarkable therapeutic efficacy in multiple cancers by suppressing key oncogenes including c-MYC [5–9]. In PDAC, several reports have demonstrated the therapeutic potential of JQ1. Mazur *et al*. reported that JQ1 suppressed PDAC tumorigenesis in genetically engineered mice by suppressing the expression of c-MYC and inflammatory cytokines including IL-6 [10]; Garcia *et al*. reported that JQ1 suppressed the growth of PDX of PDAC, though they observed minimal changes in c-Myc protein levels in most of the JQ1-treated tumors, concluding that the antitumor effects of JQ1 on PDAC were exerted through c-Myc independent mechanisms [11]; Sahai *et al*. showed that JQ1 suppressed the growth of PDAC cell lines in 3D culture irrespective of c-MYC suppression, also highlighting c-MYC-independent anti-tumor mechanisms [12]. Thus despite its promising antitumor activity, the effect of BET inhibition on CAFs and how it suppresses PDAC growth remain elusive.

In this study, we investigated the therapeutic effects of JQ1 on PDAC using PDX tumors and human CAFs. Focusing on the fact that JQ1-treated PDX tumors showed remarkable reduction in desmoplastic stroma, we demonstrated that JQ1 inactivated CAFs and suppressed their tumor promoting properties. Our data not only support the antitumor effects of JQ1 on PDAC as shown in previous reports, but also propose its new molecular mechanism through the inactivation of CAFs.

## RESULTS

### JQ1 attenuates tumor growth and desmoplasia in PDX of human PDAC

We established two PDX lines (PDX19 and PDX20) from surgically resected human PDAC tissues. Both tumors harbored mutations in codon 12 of the *KRAS* gene (Supplementary Table S1). The xenograft tumors highly recapitulated the pathology of original tumors, accompanied by abundant collagen deposition and α-smooth muscle actin (α-SMA) expressing CAFs (Supplementary Figure S1A-S1C).

**Figure 1:**
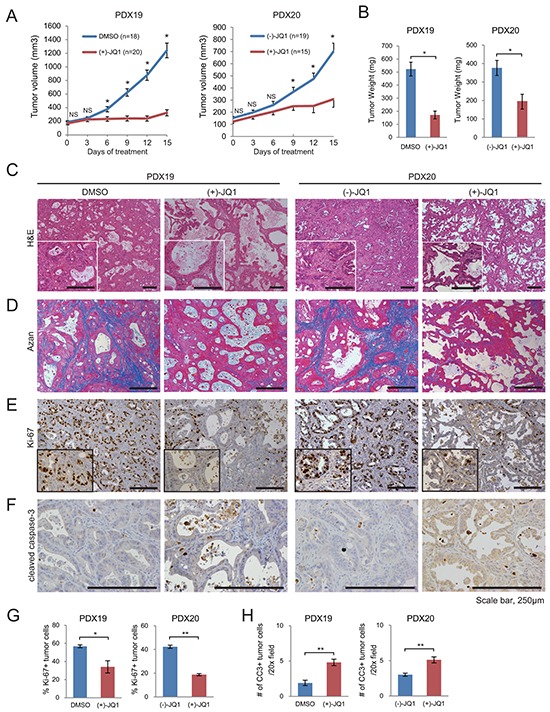
JQ1 attenuates tumor growth and desmoplasia in PDX of human PDAC Mice bearing PDX tumors were treated daily with (+)-JQ1 or control reagents (DMSO or (−)-JQ1) at 50 mg/kg for 2 wk. **A.** Average volumes of subcutaneous PDX tumors. *, *P* < .05; NS, not significant. **B.** Tumor weight at the end of the treatment period. Bars represent means ± SEM; *, *P* < .05. (C and D) H & E staining **C.** and Azan staining **D.** of PDX tumors at the end of the treatment. Scale bars represent 250 μm. Insets show higher magnification pictures. **E** and **F.** Representative IHC images stained for Ki-67 (E) and cleaved caspase-3 (CC3) (F). Scale bars represent 250 μm. Insets show higher magnification pictures. **G** and **H.** Percentage of Ki-67 (E) and CC3 (F) positive tumor cells per 20x field (average of five random fields per tumor) are shown. Four tumors per group were analyzed. Bars represent mean ± SEM (n = 4); *, *P* < .05 and **, *P* < .01.

Using these PDX models, we investigated the effects of BET inhibition. Tumor growth rates and tumor weights were significantly reduced in JQ1-treated mice compared to control mice (Figure 1A and 1B). Histologically, JQ1-treated tumors showed a marked reduction of desmoplastic stroma (Figure 1C) and fibrotic deposition, as determined by Azan staining (Figure 1D). These data demonstrate that JQ1 not only suppresses tumor growth but also attenuates desmoplastic change in PDAC. The number of Ki-67 positive tumor cells decreased significantly in JQ1-treated tumors (Figure 1E and Figure 1G). Consistently, western blotting confirmed a remarkable reduction of the proliferation markers cyclin D1 and PCNA in JQ1-treated tumors (Supplementary Figure S1D). In contrast, there was only a slight, albeit significant, increase in apoptotic cells in JQ1-treated tumors (Figure 1F and 1H). These results indicate that the antitumor effects of JQ1 on human PDAC xenograft tumors are mainly cytostatic, as described before [10].

### JQ1 exhibits minimal effects on the growth of isolated PDAC cells *in vitro*

**Figure 2:**
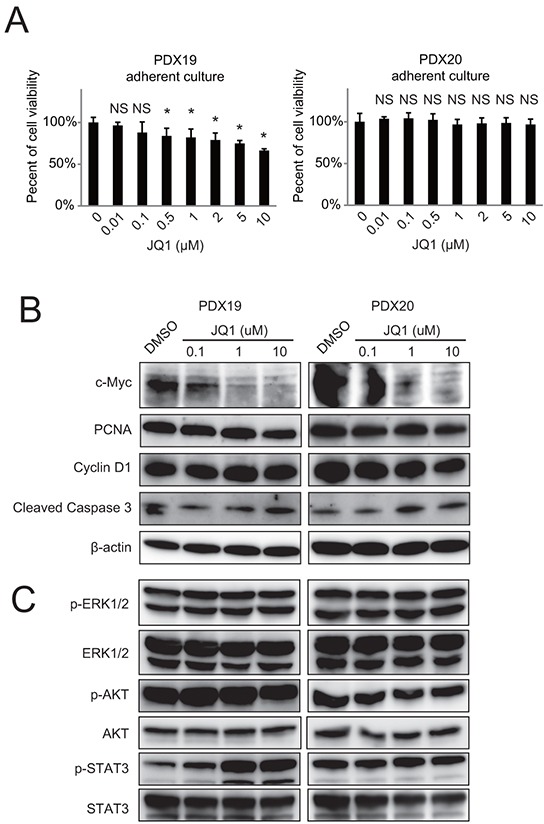
JQ1 exhibits minimal effects on the growth of primary human PDAC cells *in vitro* Primary human PDAC cells were isolated from PDX tumors and cultured under adherent conditions in DMEM/F12 containing 10% FBS. **A.** PDAC cells were incubated at indicated doses of JQ1 for 72 h, and then cell growth was quantified. *, *P* <.05 compared to vehicle by Student's *t*-test. **B** and **C.** Western blot of whole cell lysates from primary PDAC cells treated with JQ1 for 48 h *in vitro*.

To elucidate whether the cytostatic effect of JQ1 on PDAC cells was cell-intrinsic, primary PDAC cells were isolated from PDX tumors and treated with JQ1. Contrary to the remarkable growth reduction *in vivo*, JQ1 exhibited only minimal to no growth inhibitory effects in the primary PDAC cells (Figure 2A). Western blotting also confirmed that JQ1 did neither reduce PCNA and cyclin D1 expression (Figure 2B) nor affect major signaling pathways related to cell proliferation and survival (Figure 2C) in these primary PDAC cells. These differential responses of PDAC cells to JQ1 between *in vitro* and *in vivo* settings suggest that the *in vivo* tumor suppressive effects are mediated largely through a cell-extrinsic mechanism.

JQ1 reduced c-Myc protein levels in primary PDAC cells in a dose-dependent manner (Figure 2B). Although c-Myc is a key oncogene whose transcription is strongly inhibited by JQ1 in various malignancies [6–8], the *in vitro* data indicated that the *in vivo* antitumor effects of JQ1 was exerted through c-Myc independent mechanisms, as reported before [11, 12]. By contrast, JQ1 suppressed the growth of established cell lines, which was accompanied by decreased PCNA and c-Myc expression (Supplementary Figure S2). We do not exclude the possibility that the anti-proliferative effects of JQ1 for these cell lines depend on the suppression of c-Myc.

### JQ1 directly inactivates CAFs and attenuates desmoplasia in PDAC

**Figure 3:**
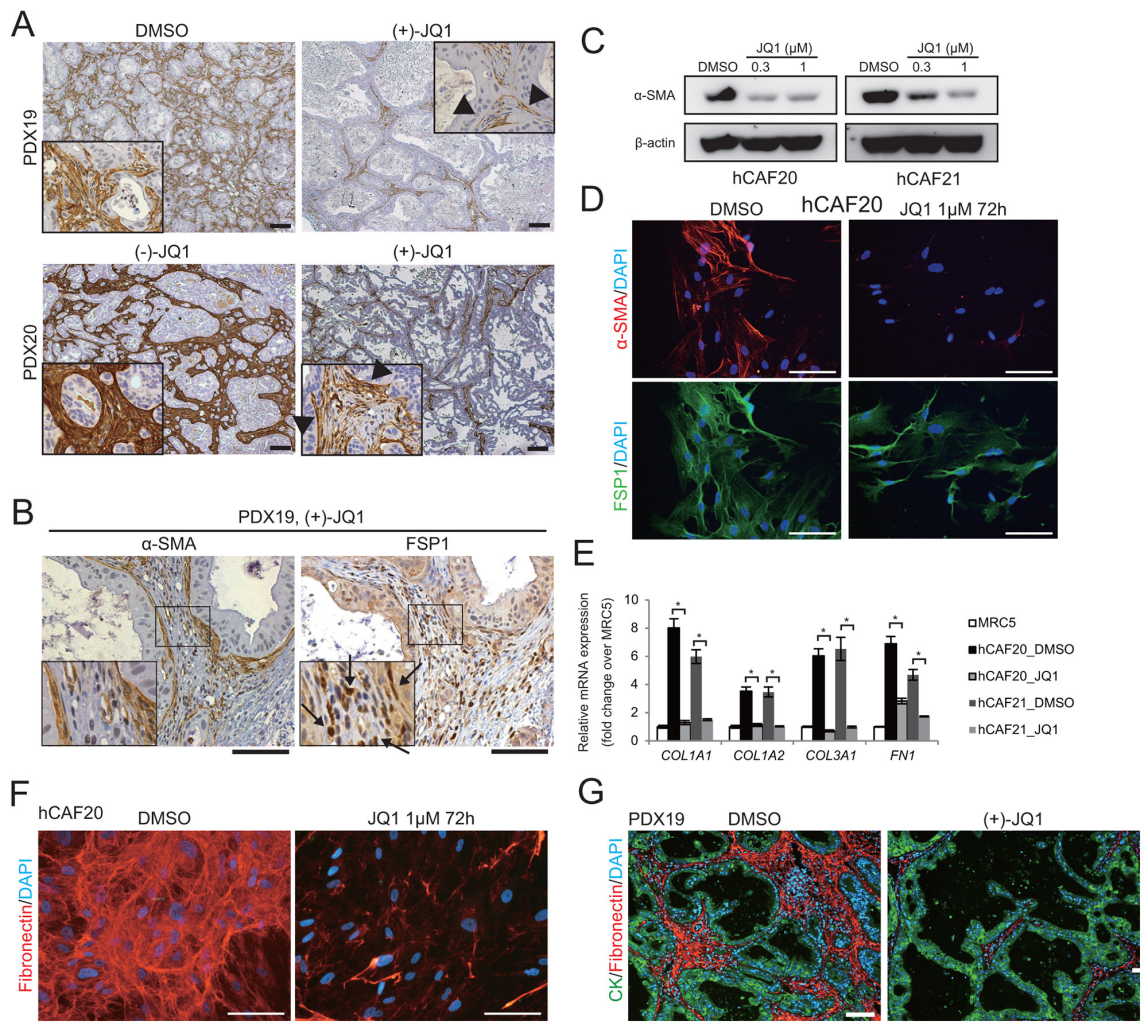
JQ1 suppresses α-SMA expression and ECM synthesis in CAFs **A.** α-SMA immunohistochemistry was performed on PDX tumors from mice treated with control reagents or JQ1 for 2 wks. In JQ1-treated tumors, positive α-SMA staining was confined to the stromal cells adjacent to the tumor epithelia (arrow heads). Scale bars represent 100 μm. **B.** Serial sections of JQ1-treated PDX tumors were stained for α-SMA and FSP1. Most stromal cells that were negative for α-SMA expression were stained positively for FSP1 (arrows), suggesting that fibroblasts not in direct contact with cancer cells lost α-SMA expression by JQ1 treatment. Scale bars represent 100 μm. **C.** Western blot of lysates from primary human CAFs (hCAF20 and hCAF21) treated with JQ1 for 48 h. JQ1 treatment reduced α-SMA expression in hCAFs in a dose-dependent manner. **D.** Immunofluorescence images of hCAF20 cells stained for α-SMA (red) and FSP1 (green) following JQ1 treatment. Nuclei were stained with DAPI (blue). Scale bars represent 100 μm. **E.** The expression levels of ECM related genes were analyzed by qRT-PCR. hCAFs were treated with 1 μM JQ1 for 48 h. MRC-5, a human lung fibroblast cell line, was used as normal fibroblasts. Values were normalized to *ACTB* and expressed as fold change against MRC-5. Bars represent means ± SEM (n = 3); *, *P* < .01, compared to respective DMSO controls. **F.** Representative immunofluorescence images of hCAF20 cells. After treatment with DMSO or 1 μM JQ1 for 72 h, hCAF20 cells were stained with antibodies against fibronectin (red). Nuclei were stained with DAPI (blue). Scale bars represent 100 μm. **G.** Representative immunofluorescence images of PDX19 tumors that were co-stained with antibodies against pan-cytokeratin (CK, green) and fibronectin (red). Nuclei were stained with DAPI (blue). Scale bars represent 100 μm.

CAF is the most dominant cell type in the PDAC stroma, playing central roles in the tumor-stromal interaction [13–15]. Immunohistochemistry revealed abundant infiltration of α-SMA expressing CAFs in the stroma of control tumors (Figure 3A). In contrast, we found a remarkable reduction of α-SMA positive cells in JQ1-treated tumors (Figure 3A). Notably, most of the α-SMA negative stromal cells in JQ1-treated tumors were positive for the fibroblast marker FSP1 (Figure 3B, arrows), suggesting that JQ1 did not eliminate CAFs but rather turned α-SMA positive CAFs into α-SMA negative ones.

To assess the direct impacts of JQ1 on CAFs, two lines of primary human CAFs (hCAF20 and hCAF21) were isolated from human PDAC tissues (Supplementary Figure S3A). Western blot analysis showed that α-SMA expression in these CAFs was diminished upon JQ1 treatment (Figure 3C). Immunofluorescence experiments also demonstrated that hCAF20 cells lost α-SMA expression following JQ1 treatment, while FSP1 expression remained unchanged (Figure 3D). JQ1 did not affect viability of CAFs unless it was used at excessive concentration (Supplementary Figure S3B). Therefore, these results suggest that that JQ1 converted α-SMA positive CAFs into α-SMA negative ones, rather than depleting CAFs.

Activated CAFs contribute to desmoplastic formation by enhanced ECM synthesis [13–15]. We hypothesized that the attenuation of PDX stroma by JQ1 was due to inactivation of the CAFs. Quantitative reverse transcriptase PCR (qRT-PCR) analysis confirmed the upregulation of ECM related genes in the isolated human CAFs compared to the normal human lung fibroblast cell line MRC-5 (Figure 3E). JQ1 treatment downregulated these ECM related genes to the level of normal fibroblasts (Figure 3E). In addition, immunofluorescence analysis confirmed reduced fibronectin production in CAFs treated with JQ1 *in vitro* (Figure 3F) and in PDX tumors treated with JQ1 *in vivo* (Figure 3G). These results indicate that JQ1 directly suppresses ECM production by CAFs in PDAC tumors (Figure 1D).

### JQ1 reduces inflammatory cytokine and growth factor secretion from CAFs

**Figure 4:**
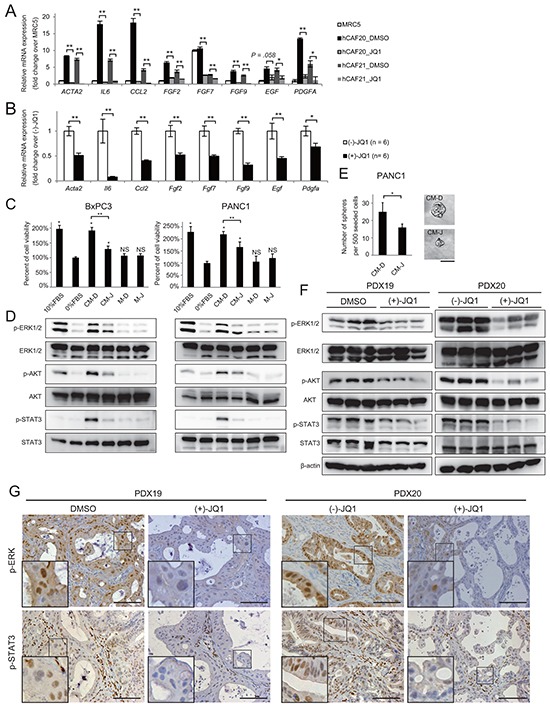
JQ1 alters the secretome of CAFs, reducing PDAC proliferation **A-B.** The expression levels of activated-CAF related genes, including inflammatory cytokines and growth factors, were analyzed by qRT-PCR in hCAFs *in vitro* (A) and in the stromal cells of PDX tumors *in vivo* (B). (A) hCAFs were treated with 1μM JQ1 for 48 h. Values were normalized to *ACTB* and expressed as fold change over MRC-5. For *FGF7*, the value of MRC5 was set to 10. Bars represent means ± SEM (n = 3); *, *P* < .05 and **, *P* < .01, compared to the respective DMSO controls. (B) Bulk RNA from PDX20 tumors treated with (−)-JQ1 or (+)-JQ1 for 2 weeks were used for qRT-PCR. Bars represent means ± SEM (n = 6); *, *P* < .05 and **, *P* < .01, compared to (−)-JQ1-treated tumors. **C-D.** CM from hCAF20 cells enhances pancreatic cancer cell proliferation, accompanied by the activation of ERK, Akt, and STAT3 pathways. CM-D and CM-J represent CM collected from hCAF20 cells treated with DMSO or JQ1, respectively. Negative control medium without CAF culture was prepared in the same manner either with DMSO or JQ1 (referred to as M-D and M-J). **C.** The effects of CM on PDAC cell proliferation. BxPC3 and PANC1 cells were cultured under the indicated conditions for 72 h, and viable cells were quantified. **D.** Western blot was performed using lysates from cells after 15 min incubation with the indicated CM. **E.** A tumor sphere formation assay was performed. Bars represent means ± SEM (n = 5); *, *P* = .007. Representative images of tumor spheres are shown. Bars represent 100 μm. **F.** Western blot using whole lysates from PDX tumors treated with either JQ1 or control reagents. Analyses of three tumors from each group are shown. **G.** Representative immunohistochemistry images stained for p-ERK and p-STAT3. Positive p-ERK staining was observed exclusively in cancer cells, while positive p-STAT3 staining was observed both in cancer cells and stromal cells. Scale bar represents 250 μm.

Enhanced cytokine and growth factor secretion is another key feature of the activated CAFs. Conditioned media (CM) from hCAF20 cells that were pre-treated with DMSO or JQ1 (denoted as CM-D and CM-J, respectively) was applied to cytokine array. Various inflammatory cytokines including interleukin-6 (IL-6) and CCL2 that are upregulated in activated CAFs [13, 16, 17] were significantly decreased in the CM-J as compared to CM-D (Supplementary Figure S4). qRT-PCR also demonstrated that JQ1 decreased *ACTA2*, *IL6*, and *CCL2* mRNA expression to the level of normal fibroblasts (Figure 4A). Moreover, these CAFs also showed increased expression of growth factors including fibroblast growth factors (FGFs), epidermal growth factor (EGF) and platelet derived growth factor (PDGF) (Figure 4A), which have been reported to be upregulated in activated CAFs and contribute to cancer proliferation [1, 13, 15]. JQ1 also downregulated the expression of these growth factors (Figure 4A).

In the establishment of PDX tumors, mouse stroma cells replace human-derived stromal cells [18]. Therefore, qRT-PCR using mouse specific primers reveal mRNA expression in the stromal cells of PDX tumors [18]. Consistent with the *in vitro* data, activated-CAF related genes were downregulated in the stromal cells of JQ1-treated PDX tumors (Figure 4B), suggesting that JQ1 treatment directly suppressed the production of cytokines and growth factors from CAFs both *in vitro* and *in vivo*.

### JQ1 alters the secretome of CAFs, reducing PDAC proliferation

Our data suggest that JQ1 exerts *in vivo* anti-tumor effects through cell-extrinsic mechanisms, rather than directly inhibiting PDAC cell proliferation. Thus, we hypothesized that the altered secretome of CAFs treated with JQ1 suppressed PDAC cell proliferation in the PDX tumors. To confirm this, PDAC cells were cultured with CM-D and CM-J. As reported before [17, 19], CM-D promoted the growth of PDAC cells (Figure 4C) accompanied by activation of ERK, AKT, and STAT3 (Figure 4D). In contrast, this capacity of CM-D was diminished in CM-J (Figure 4C and 4D). Moreover, a tumor sphere formation assay, which indicates *in vivo* tumor-initiating potential [20], demonstrated that CM-D induced tumor sphere formation from PANC1 cells without growth factor supplementation. As expected, CM-J showed decreased potential to induce tumor spheres (Figure 4E). These results indicate that CAFs secrete soluble factors that promote PDAC growth, which was suppressed by JQ1 treatment. Importantly, reduced activation of those signaling pathways was also observed in JQ1-treated PDX tumors (Figure 4F and 4G). Along with the data that JQ1 did not affect signaling pathways in isolated PDAC cells *in vitro* (Figure 2C), these results emphasize that the tumor-promoting secretome from CAFs contributes to *in vivo* PDAC growth, which is suppressed by JQ1.

### BRD4 knockdown recapitulates the effects of JQ1 on CAF inactivation

Among BET family members, BRD4 is most selectively inhibited by JQ1. Thus, to determine whether JQ1 inactivates CAFs in a BRD4-dependent manner, small interfering RNA (siRNA)-mediated knockdown of *BRD4* was performed. Similar to the effects of JQ1, *BRD4* knockdown downregulated α-SMA expression (Supplementary Figure S5A) and other genes related to activated CAFs (Supplementary Figure S5B). BRD4 knockdown did not reduce the expression of these genes as effectively as JQ1, suggesting the involvement of other BET proteins. Nevertheless, these results indicate that the inactivation of CAFs by JQ1 is mediated, at least in part, through antagonistic effects against BRD4.

### JQ1 inhibits Hedgehog and TGF-β pathways, potent regulators of CAF activation

It has been reported that BRD4 associates with virtually all active promoters and also with a considerable fraction of enhancers [9, 21]. To elucidate the mechanism of how JQ1 inactivates CAFs, we focused on the gene expression regulated by Hh and TGF-β pathways, two major activators of CAFs in desmoplasia formation [22–25].

**Figure 5:**
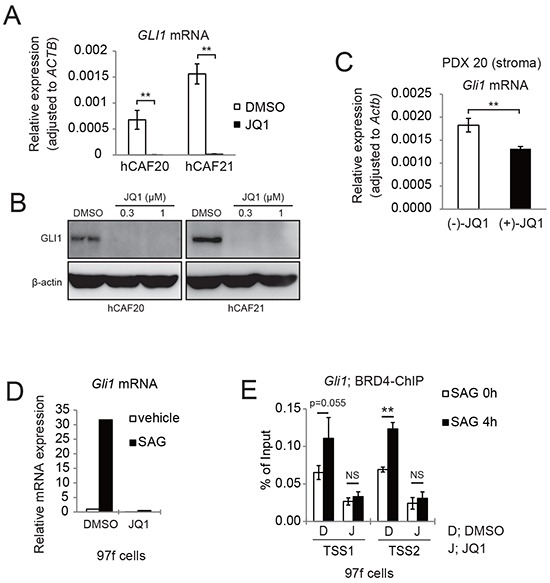
JQ1 inhibits Hh target gene GLI1 expression in both human and murine CAFs **A.** qRT-PCR analysis showed that JQ1 reduced *GLI1* mRNA expression in human CAFs. hCAFs were treated with DMSO or 1μM JQ1 for 24 h. Bars represent means ± SEM (n = 3); **, *P* < .01, compared to DMSO treated controls. **B.** Western blots showed that JQ1 reduced GLI1 expression in human CAFs. hCAFs were treated with DMSO or JQ1 for 24 h. **C.** qRT-PCR showed that *Gli1* mRNA expression was reduced in murine stromal cells of PDX20 tumors that were treated with (+)-JQ1 for 14 days. Bars represent means ± SEM (n = 6); **, *P* < .01, compared with (−)-JQ1-treated tumors. **D.** qRT-PCR analysis showing that JQ1 reduced *Gli1* mRNA expression in murine CAFs (97f cells). 97f cells were treated with DMSO (D) or 1μM JQ1 (J) for 2 hours, followed by addition of DMSO or 0.3 μM SAG for 24 h. **E.** ChIP-qPCR revealed that SAG-induced BRD4 recruitment to *Gli1* promoters were suppressed by JQ1 treatment. After pretreatment with DMSO (D) or JQ1 (J) for 2 h, 97f cells were stimulated with SAG for 0 or 4 h and subjected to ChIP-qPCR. Bars represent means ± SD (n = 3); *, P < .05; **, *P* < .01.

GLI1 is a major target gene of the Hh pathway, and it has been reported that BRD4 directly regulates *GLI1* transcription in mouse embryonic fibroblasts [26]. We found that JQ1 reduced *GLI1* mRNA and protein levels in human primary CAFs (Figure 5A and 5B), and also observed reduced *Gli1* mRNA expression in the stroma of JQ1-treated tumors (Figure 5C). These results indicate that JQ1 affects GLI1 expression in CAFs both *in vitro* and *in vivo*. We also found that Smoothened agonist (SAG) [27] upregulated *Gli1* mRNA expression in mouse CAF 97f cells, which was inhibited by JQ1 pretreatment (Figure 5D), indicating that JQ1 inhibits the canonical Hh pathway. Consistently, ChIP-qPCR confirmed that JQ1 disrupted Brd4 binding to the *Gli1* promoter regions under SAG-mediated Hh signaling activation (Figure 5E).

**Figure 6:**
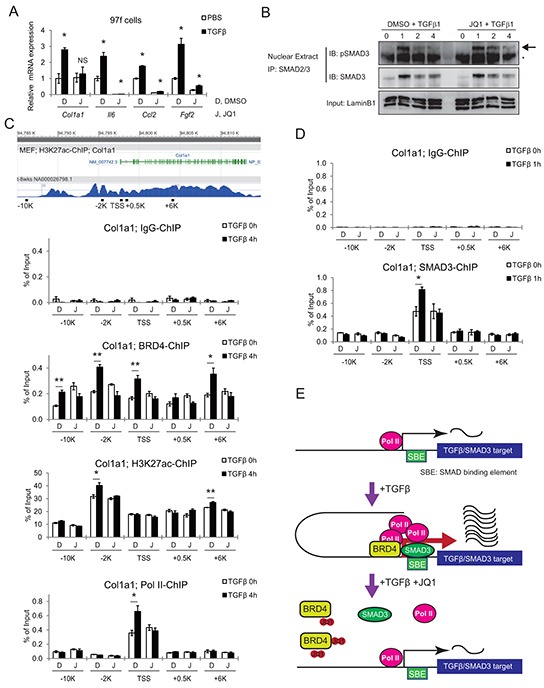
JQ1 inhibits the TGF-β pathway by disrupting BRD4 binding to promoter regions of target genes **A.** qRT-PCR shows TGF-β-induced upregulation of activated CAF related genes, which was suppressed by JQ1 pre-treatment. Mouse CAF (97f) cells were pretreated with DMSO (D) or 1 μM JQ1 (J) for 2 h, followed by TGF-β1 (10ng/mL) stimulation for 24 h. Values were normalized to *Actb* and expressed as fold change against control. Bars represent means ± SEM (n = 3); *, *P* < .05, compared to respective DMSO controls. **B.** JQ1 did not affect phosphorylation or nuclear translocation of Smad3. Nuclear extracts were isolated from 97f cells after pretreatment with DMSO or 1 μM JQ1 followed by TGF-β1 (10ng/mL) stimulation for the indicated times. Nuclear extracts were immunoprecipitated with an anti-Smad2/3 antibody followed by immunoblot. The arrow indicates bands for pSmad3 and the asterisk indicates nonspecific bands. **C.** Tracks are H3K27ac ChIP-seq data from MEF (ENCODE). Schematic representations of mouse *Col1a1* genes and ChIP primers are shown. Localization of primers is depicted as distances from the TSS. After pretreatment with DMSO (D) or JQ1 (J) for 2 h, 97f cells were stimulated with TGF-β1 (10ng/mL) for 0 or 4 h and subjected to ChIP-qPCR. **D.** SMAD3-ChIP was also performed on 97f cells after TGF-β stimulation for 0 or 1 h. Bars represent means ± SEM (n = 3); *, *P* < .05. **E.** Model schematic for JQ1-mediated inhibition of TGF-β/Smad3 gene transcription.

TGF-β is another well-known stromal activator in various cancers [28, 29]. To address the role of BET inhibition in the TGF-β pathway, CAFs were pretreated either with DMSO or JQ1 for 2 h after 24 h serum starvation, followed by stimulation with TGF-β. qRT-PCR analysis showed upregulation of several activated-CAF related genes, which was suppressed by JQ1 pretreatment (Figure 6A). In this setting, phosphorylated Smad3 translocated into the nucleus in 1 h, and promptly became undetectable after 3 h (Figure 6B). Importantly, JQ1 did not significantly affect TGF-β-induced phosphorylation and subsequent nuclear translocation of Smad3 (Figure 6B). We chose *Col1a1* [17, 30] and *Il6* as representatives of profibrogenic and proinflammatory genes in the TGF-β pathway. ChIP-qPCR revealed that TGF-β significantly increased Brd4 occupancy at the promoter regions of both genes (Figure 6C and Supplementary Figure S6). TGF-β-induced recruitment of Brd4 was highest at −2 kb from the *Col1a1* transcriptional start site (TSS) and +0.6 kb from the *Il6* TSS, where acetylated chromatin (H3K27ac) level was also the highest. Corresponding to these changes, evident enrichment of RNA polymerase II (Pol II) was observed at the TSS of *Col1a1* and +0.2 kb from the TSS of *Il6*. These TGF-β-induced changes were completely blocked by JQ1 pretreatment.

**Figure 7:**
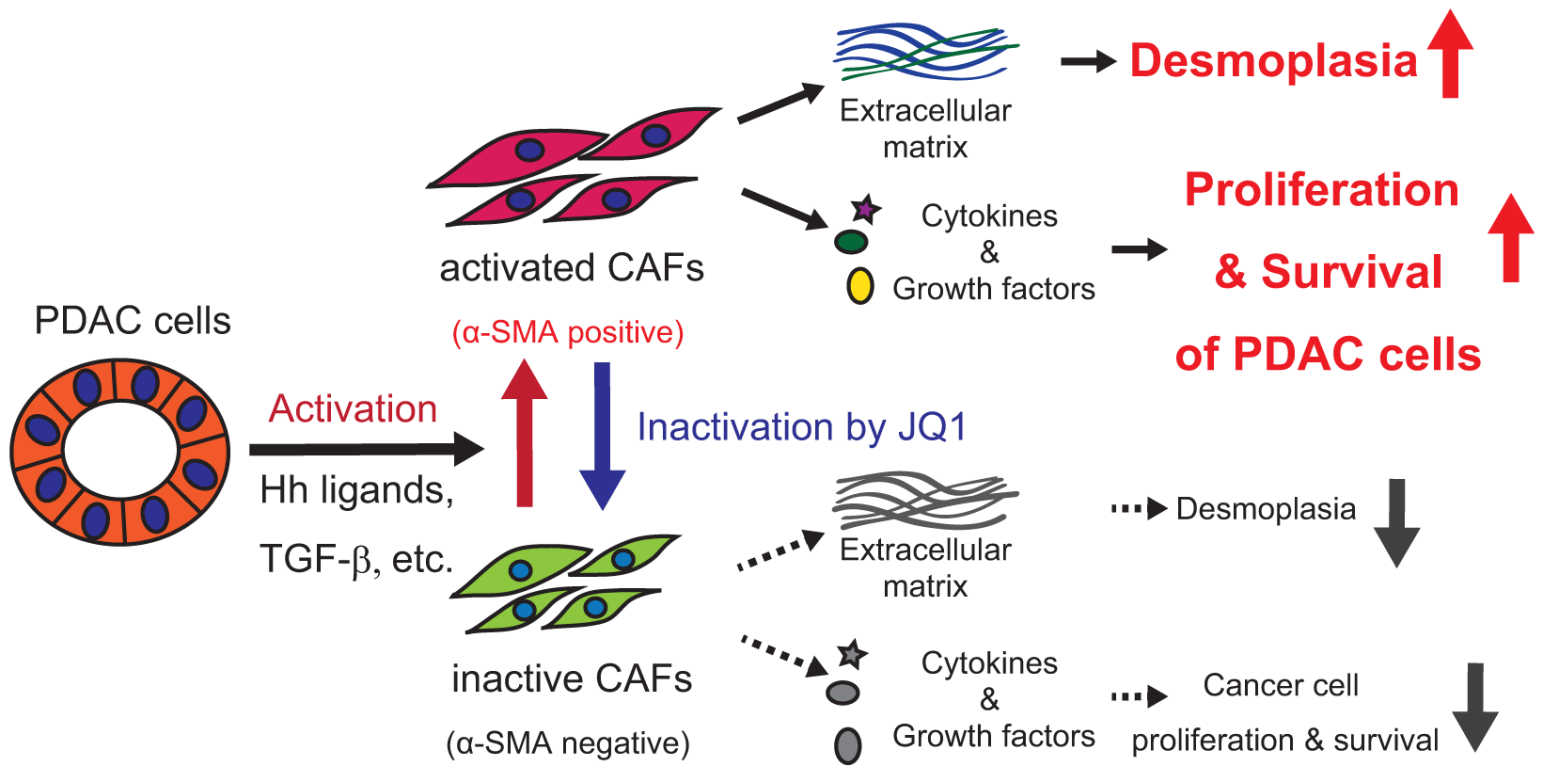
Graphical summary

ChIP-qPCR revealed a Smad3 binding site around the TSS of the *Col1a1* gene, where a significant increase of Smad3 binding was observed following TGF-β treatment (Figure 6D). Notably, the consensus Smad binding element (SBE) sequence, a GTCTAGAC motif, was found at +92 to +99 bases downstream of the *Col1a1* TSS. Interestingly, this TGF-β-mediated recruitment of Smad3 was disrupted by JQ1 pretreatment (Figure 6D). These findings suggested that BET inhibition disrupted the recruitment of transcriptional machinery including transcription factor at the target genes (Figure 6E). Therefore, these data indicate that JQ1 inhibits two major regulators of CAF activation, Hh and TGF-β pathways, at the transcriptional output (Figure 7).

### The combination of JQ1 and gemcitabine showed additional efficacy over gemcitabine monotherapy

Our data indicated that JQ1 exerts anti-tumor effects mainly through the modulation of the tumor-microenvironment. Hypothesizing that the combination of JQ1 and the cytotoxic agent gemcitabine, two drugs with distinct anti-tumor activities, may confer therapeutic benefits, we treated PDX with gemcitabine alone or gemcitabine plus JQ1. The combination therapy showed additional efficacy over gemcitabine monotherapy, as demonstrated by significant reduction in subcutaneous tumor growth rate and tumor weights (Supplementary Figure S7A and S7B). Moreover, compared to tumors treated with gemcitabine-monotherapy, combination-treated tumors showed significant reduction in the proliferation marker Ki-67 (Supplementary Figure S7C) as well as a significant increase in the apoptotic marker cleaved caspase-3 (Supplementary Figure S7D). These data, along with recent reports [10–12], support the therapeutic potential of BET inhibition in human PDAC.

## DISCUSSION

So far several studies have reported a therapeutic efficacy of BET inhibition on PDAC [10–12], though underlying mechanisms of antitumor efficacy and how BET inhibition affects CAFs remain elusive. Using PDX models and primary cultures of CAFs, we identified critical roles for BET proteins in CAF activation and demonstrated that the inactivation of CAFs by BET inhibition suppressed PDAC growth in a paracrine manner.

In this study, JQ1 remarkably suppressed the growth of PDX tumors *in vivo*, while only minimal effects were observed on isolated PDAC cells *in vitro*. This discrepancy does not deny any direct tumor-suppressive effects of JQ1 *in vivo* that were described by other groups [10, 11]. Indeed, given the fact that JQ1 strongly suppressed the growth of established PDAC cell lines in the same culture conditions (Supplementary Figure S2), it is possible that the patient-derived cancer cells we used are inherently resistant to JQ1 treatment. More importantly, this difference indicates that even in tumors where cancer cells do not respond to JQ1, JQ1 can still suppress tumor growth via modulation of the tumor microenvironment.

Two recent studies reported therapeutic effects of CAF-inactivation via retinoic acid (RA) [31] and vitamin-D receptor (VDR) signaling [17]. Similarly, JQ1 downregulated multiple key genes in CAFs, including *ACTA2*, a marker of activated CAFs; fibrotic genes including *COL1A1* and *FN1* [32]; pro-inflammatory cytokines including *IL6* [33] and *CCL2* [34]; and growth factors including PDGF, FGFs, and EGF [1, 13], resulting in similar phenotypic changes. Furthermore, a recent report classified PDACs according to the expression profiles of stromal components into two subtypes, normal and activated stromal subtypes, the latter of which is associated with worse prognosis [35]. Notably, *COL1A1* and *FN1* are among the most highly expressed genes in the activated stroma subtype [35], suggesting tumor-promoting roles of activated CAFs. Thus our data, along with previous studies, support the concept of normalizing CAFs as a promising therapeutic approach [36]. Interestingly, a very recent study reported critical roles of BRD4 in liver fibrosis, demonstrating that JQ1 suppressed the activation of hepatic stellate cells and inhibited CCl4-induced liver fibrosis [37]. Given that CAFs and HSCs harbor no genetic mutations [38], targeting non-tumor cells by epigenetic modulators may be a reasonable strategy.

In this analysis, we revealed that JQ1 suppresses Hh and TGF-β pathways, two major pathways implicated in CAF activation, though it is unclear whether other pathways are also affected and to what extent these two pathways contribute to the phenotypic changes. We confirmed that JQ1 inhibits *GLI1* transcription by preventing BRD4 from binding to the promoter regions, as described before [26]. We also revealed that JQ1 inhibits TGF-β/SMAD signaling. Importantly, JQ1 not only inhibited BRD4 and Pol II binding to the promoter regions and TSS, but also disrupted TGF-β-induced SMAD3 binding to the SBE within the promoter of the *Col1a1* gene (Figure 6E). Though it is not clear whether JQ1 inhibits TGF-β-induced SMAD3 recruitment globally or only to a fraction of SMAD3-target genes, this is an unexpected result given the previous report showing that TNF-α-induced p65 recruitment was not affected by BET inhibition [39]. One possibility is that JQ1 might disrupt the local chromatin structure and make SBE inaccessible to Smad3, possibly through the disruption of the enhancer-promoter interaction. Another possibility is that BRD4 may act as a DNA-binding cofactor and strengthen the interaction between Smad3 and DNA, like TRIM33, which interacts with both Smad3 and specific histone modifications [40].

In contrast to the evidence supporting stroma-targeting therapy, recent clinical trials using the Hh inhibitor saridegib in combination with gemcitabine revealed shorter patient survival than that achieved with gemcitabine alone. Likewise, evidence shows that the suppression of desmoplasia through Hh inhibition accelerated tumor growth and decreased survival in murine models of PDAC [41, 42]. This is quite different from RA- or VDR-mediated inactivation of CAFs [17, 31]. These differences indicate that stromal-targeting therapies may result in different outcomes depending on the signaling pathways that are modulated. Given that JQ1-induced inactivation of CAFs is, at least in part, mediated through the suppression of both Hh and TGF-β pathways, further investigation is required to determine the long-term effects before BET inhibition can be applied to clinical settings. It would also be important to study the effects on other stromal cells. Indeed, we observed a reduced macrophage infiltration in JQ1-treated tumors (Supplementary Figure S8). Moreover, our data suggested the possibility that JQ1-mediated inactivation of CAFs impairs macrophage recruitment and also affects macrophage function (Supplementary Figure S8).

Importantly, while recent reports pointed to tumor-suppressive roles of desmoplasia and CAFs in PDAC [41–43], preclinical evidence also showed that the reduction of desmoplasia can improve accessibility to cancer cells and enhance antitumor response to cytotoxic chemotherapeutic agents [17, 44, 45], anti-VEGFR therapy [41], and immunotherapy [43, 46, 47]. In this regard, our next aim would be to optimize the therapeutic strategies in combination with BET inhibition for PDAC.

## MATERIALS AND METHODS

### Human pancreatic tissues

Pancreatic tissues were obtained from patients who underwent pancreatectomy for pancreatic cancer at the University of Tokyo Hospital. Written informed consent was obtained from each patient, and the Ethical Committee for Clinical Research of our institution approved this study. The clinicopathological characteristics of primary pancreatic cancer specimens are described in Supplementary Table S1.

### Primary tumor xenografts

All animal experiments were carried out using protocols approved by the Animal Ethics Committee of the University of Tokyo. Primary PDAC xenografts were established as previously described [48]. Freshly resected human PDAC specimens were obtained from pancreatic cancer patients who underwent pancreatectomy. Briefly, tumor fragments were suspended in DMEM/F12 with 2% penicillin, streptomycin, and amphotericin, and were mechanically minced with scissors. Tumor fragments were implanted into the subcutaneous spaces in the back of five to eight-week-old male NOD/SCID mice (CLEA Japan, Inc.). When tumors reached 1,000-2,000 mm3, the mice were sacrificed, and tumors were harvested and minced with razor blades. These tumor fragments were then re-transplanted into new mice or suspended in CELLBANKER 1 (ZENOAQ) and stored in liquid nitrogen for later implantation. All experiments were performed using passage 3 to 6 xenografts.

### Preparation of tumor cell suspension from PDX tumor

Harvested PDX tumors were placed on a 10 cm dish containing 5 mL DMEM/F12 with 2% antibiotics (penicillin, streptomycin, and amphotericin), and were mechanically minced using sterile razor blades. Minced tumors were then enzymatically digested with DMEM/F12 supplemented with 2% antibiotics, Collagenase IV (100 U/mL, Invitrogen), Hyaluronidase IV (0.05mg/mL, Sigma), Dispase (0.5 U/mL, STEMCELL Technologies, #07913), and DNase I (0.1 mg/mL, Roche) at 37°C for 1 h. Then, 10% fetal bovine serum (FBS) was added to inactivate the enzyme. The tumor cell suspension was sieved sequentially through a metal mesh tea strainer and a 100 μm nylon mesh, and was washed twice with DMEM/F12 supplemented with 2% antibiotics. This tumor suspension contains both cancer cells and stromal cells.

### Isolation and *in vitro* culture of primary PDAC cells

For the isolation of primary PDAC cells, whole tumor cell suspensions, as prepared above, were centrifuged at 100 ×*g* for 1 min and the supernatant was removed. The pellet was further washed twice with PBS followed by centrifugation at 100 ×*g* for 1 min (most of the stromal components and hematopoietic cells were depleted at this differential centrifugation step), and the resultant pellet was enriched for epithelial organoids composed of cancer cells. These PDAC cells were cultured under adherent conditions in collagen-coated dishes in DMEM/F12 with 2% antibiotics and 10% FBS. To remove remaining fibroblasts or other stromal cells, partial trypsinization was performed.

### *In vivo* treatment of PDX tumor with JQ1

Male NOD/SCID mice (5-7 week old) were inoculated with tumor cell suspensions (prepared as described in Supplementary Methods) into the subcutaneous tissue using 25-gauge needles. When PDX tumors reached a size of approximately 200 mm^3^, typically 2-4 weeks after tumor implantation, mice were randomized into respective treatment groups. Drug administration started on day 1 and ended on day 15. Tumor size was measured every three days from day 0 to day 15. Tumor volume was calculated by the following formula: Width x Height x Length x 0.52. After 15 days of treatment, mice were sacrificed and tumors were harvested. The BET bromodomain inhibitor (+)-JQ1 [3] (also denoted as “JQ1”) and its inactive enantiomer (−)-JQ1 was dissolved in DMSO at a concentration of 100 mg/ml and aliquoted for frozen stocks. Working solutions were prepared by diluting at 1:25 ratio (4% DMSO) in corn oil and were administered at 50 mg/kg daily by i.p. injection. Gemcitabine was administered twice a week (75 mg/kg i.p.) on days 1, 5, 8, 12, and 15.

### Isolation of human CAFs

CAFs were isolated as previously described [19, 24]. Briefly, fresh pancreatic cancer tissue was minced with a razor blade and seeded on a collagen-coated culture dish in the presence of DMEM High Glucose (Sigma) supplemented with 10% FBS (Invitrogen) and 2% penicillin, streptomycin, and amphotericin. When fibroblasts grew up to confluence, cells were trypsinized and passaged at 1:2-1:3. Purity of isolated fibroblasts was assessed by morphology and immunohistochemistry for αSMA and fibroblast-specific protein-1 (FSP1). All experiments were performed using fibroblasts under 10 passages.

### Cell lines and culture conditions

BxPC-3, PANC1, and MRC-5 cells were obtained from American Type Culture Collection (ATCC) and passaged in our laboratory for fewer than 6 months after resuscitation. These cell lines, human CAFs, and murine CAF (97f) cells were maintained in high glucose DMEM (Sigma) supplemented with 10% FBS (Invitrogen) and 1% penicillin and streptomycin (Invitrogen) at 37°C in a 5% CO_2_ humidified incubator. When used for assays, CAFs were cultured in media containing 0.5% FBS and otherwise the same components.

### Cell viability and proliferation assay

Isolated primary PDAC cells or human CAFs were seeded at 2 × 10^3^ to 4 × 10^3^ cells per well in collagen-coated 96-well plates. On the following day, JQ1 or DMSO was added at indicated concentrations. After 72 h, viable cells were quantified using Cell Counting Kit-8 (Dojindo), according to the manufacturer's protocol. To evaluate the effects of CAF-conditioned media (CM) on cancer cell proliferation, BxPC-3 and PANC1 cells were seeded at 4 × 10^3^ cells per well in collagen-coated 96-well plates and cultured in serum-free DMEM for 24 h. Concentrated CM was added for a final concentration of 20%. FBS or serum-free media was added to control samples. After 72 h, viable cells were quantified using the Cell Counting Kit-8 (Dojindo) according to the manufacturer's protocol.

### Preparation of conditioned media

Human or mouse CAFs (hCAF20 cells or 97f cells) were plated at a density of 1 × 10^6^ cells per 10 cm dish in DMEM supplemented with 10% FBS. On the next day, the media were changed to DMEM supplemented with 0.5% FBS and DMSO (0.01%) or 1 μM JQ1. After 48 h incubation, the media was changed to 4 mL serum-free, phenol red-free DMEM with DMSO or JQ1 and incubated for 24 h. For preparation of the negative control media without CAFs, serum-free DMEM with DMSO or JQ1 was incubated for 24 h in empty dishes. After incubation, the culture supernatant was collected, and filtered through a 0.45 μm filters. For cytokine array, these conditioned media (CM) were evaluated without further processing. For other experiments, 36 mL of culture supernatant was concentrated with Amicon Ultra-15 Centrifugal Filter Devices (for 3,000 NMWL) by centrifuging at 3,500 rpm for 2 h. At this step, molecules with molecular weights less than 3000, including JQ1, were diafiltrated and removed. The typical volume after concentration was 200 μL, and each concentrated media was further diluted and adjusted to 1.2 mL. Aliquots were stored at −80°C until use. For the evaluation of signal pathway activation, BxPC-3 and PANC1 cells were seeded at 1 × 10^5^ cells per well in 12-well plates and cultured in serum-free medium for 24h. Media was changed to serum-free DMEM containing 20% concentrated media. FBS (10%) and serum-free DMEM were added to control cells. After 15 min, cells were harvested and analyzed by western blot.

### Tumor sphere formation assay

Tumor sphere formation was performed as previously described [20] with slight modifications. Briefly, cells were cultured in suspension in DMEM (Sigma) supplemented with B27 (1:50, Invitrogen), 1% methylcellulose (Sigma), and 20% CM. A total of 500 cells were plated into 24-well Ultra Low Attachment plates (Costar), and spheres with a diameter larger than 70 μm were counted after 10 days of culture.

### Histology and immunohistochemistry

PDX tumors were harvested and processed as previously described [49]. The slides were hematoxylin & eosin (H&E) stained and subjected to histological analysis. Immunohistochemistry (IHC) was performed as described before [50] using VECTASTAIN Elite ABC kit (Vector Laboratories). Antibodies used and antigen-retrieval methods are shown in Supplementary Table S2. For quantification, the number of tumor cells with positive Ki-67 and cleaved caspase-3 staining were counted per 20X field. For both analyses, five random 20x fields per tumor were captured and examined. For Ki-67 staining, the total number of tumor cells per field was also counted, and results are represented as the percentage of Ki-67 positive tumor cells per field. Typically, necrotic tissues and tumor cells undergoing anoikis, which are observed in the lumens of tumor ductal structures, are stained positive for cleaved caspase-3 [45], and these cells were not counted in the quantification.

### Western blot analysis

Whole cell lysates were prepared by lysing PDX tumors with RIPA buffer (10mM Tris-HCl (pH 7.4), 150mM NaCl, 2mM EDTA, 1% NP40, 0.1% Na deoxycholate, 0.1% SDS, 50mM NaF, 1mM Na3VO4, protease inhibitor cocktail (Complete Mini, Roche)) using a homogenizer. The lysates were sonicated for 5 min using a sonicator (Bioruptor UCD-250, Cosmo bio), centrifuged at 15,000 ×*g* for 15 min at 4°C, and supernatants were collected. Protein concentrations were determined using the Bio-Rad Protein Assay (Bio-Rad). Whole cell lysates of cultured cells were prepared by directly adding Laemmli buffer. Nuclear extract was isolated from 97f cells as previously described [50]. Nuclear extract was immunoprecipitated using anti-SMAD2/3 antibody (Santa Cruz, sc-133098) followed by SDS-PAGE and immunoblot (IB) using anti-SMAD3 and anti-phospho SMAD3. Immunoblot was performed as previously described [50]. The primary antibodies used are listed in Supplementary Table S2. A horseradish peroxidase-conjugated secondary antibody (Amersham Biosciences) was used at a 1:5,000 dilution. Protein–antibody complexes were detected using ECL Plus (Amersham Biosciences).

### Quantitative reverse transcriptase PCR (qRT-PCR)

Total RNA was isolated from PDX tumors using Isogen-II (NIPPON GENE) according to the manufacturer's protocol, and total RNA was extracted from cultured cells using NucleoSpin RNA II (TAKARA). Isolated RNA was DNase treated and reverse transcribed using the ImProm-II Reverse Transcription System (Promega). qRT-PCR was performed on the StepOnePlus Real-Time PCR System (Applied Biosystems) using the FastStart Universal SYBR Green Master (Roche). Each assay was performed using biological samples in triplicate. The relative amount of gene transcripts was evaluated by the Ct method and normalized to that of *ACTB* or *Actb*. The primers used for qRT-PCR are shown in Supplementary Table S3.

### Immunofluorescence

CAFs were seeded on a 4-well Lab-Tek Chamber Slide (Thermo, 177399) at a density of 5 × 10^4^ cells/500 μL medium per well. After incubation with JQ1 for indicated times, cells were fixed with 2% paraformaldehyde, permeabilized with 0.25% TritonX-100, and incubated with primary antibodies. For immunofluorescence, Alexa Fluor 488 goat anti-rabbit and Alexa Fluor 555 goat anti-mouse were used as secondary antibodies (Life Technologies), and DAPI was used for nuclear staining. The primary antibodies used are listed in Supplementary Table S2.

### Chromatin immunoprecipitation (ChIP) assay

For chromatin immunoprecipitation (ChIP) assay, we used mouse CAF cells (97f) that were isolated from *Ptf1a-^Cre/+^*; LSL-Kras*^G12D/+^*;Tgfbr2*^flox/flox^* mice, a genetically engineered mouse model of PDAC that well recapitulates desmoplastic stroma in human PDAC [49]. For each assay, sheared chromatin equivalent to 7.5 × 10^6^ cells were used. For each assay, 40 μL of magnetic beads (Dynabeads M-280 Seep anti-Rabbit IgG, Life Technologies) were blocked with 0.5 % BSA in PBS and further bound with 4 μg of indicated antibodies overnight at 4°C. Antibodies used for ChIP were: BRD4 (Bethly A301-985A), H3K27ac (Abcam, ab4729), RNA polymerase II (Santa Cruz, sc-899X), SMAD3 (Abcam, ab28379), and normal Rabbit IgG (Cell Signaling Technology, CST2729). After purification of precipitated DNA, qPCR was performed. Detailed procedures for ChIP are described in Supplementary Methods. The ChIP primers are listed in Supplementary Table S4.

### Statistical analysis

Data represents means ± SEM. The significance of differences between experimental groups was evaluated using the two-sided Student's *t*-test. Significance was defined as *P* < .05.

## SUPPLEMENTARY DATA, FIGURES AND TABLES






