# Supplementary material for: Stromal remodeling by the BET bromodomain inhibitor JQ1 suppresses the progression of human pancreatic cancer
Source: Oncotarget. 2016 Aug 9;7(38):61469–84. doi: 10.18632/oncotarget.11129 (PMC5308665; doi:10.18632/oncotarget.11129)
Supplement: Supplementary file 1 [file oncotarget-07-61469-s001.pdf]

Stromal remodeling by the BET bromodomain inhibitor JQ1 suppresses the progression of human pancreatic cancer

SUPPLEMENTARY DATA

SUPPLEMENTARY RESULTS

It is also important to clarify the effects on other stromal cells. For example, within the tumor stroma are abundant immunosuppressive myeloid cells including tumor-associated macrophages (TAMs). TAMs have been implicated in the suppression of anti-tumor immunity, promotion of cancer cell proliferation, and treatment resistance in PDAC [1]. In fact, an increased number of TAMs within the tumor has been shown to correlate with poor prognosis in PDAC patients [2]. Interestingly, we observed reduced infiltration of macrophages in JQ1-treated PDX tumors (Supplementary Figure S8A).

The chemokine CCL2 plays a central role in the mobilization and recruitment of CCR2-positive inflammatory monocytes from the bone marrow to the blood and finally into the tumors [3]. So far several studies have reported therapeutic efficacy of targeting the CCL2-CCR2 chemokine signaling axis in PDAC [4]. Based on the significant reduction of *CCL2* expression in JQ1-treated CAFs (Figure 4A), we hypothesized that an altered secretome from CAFs might contribute to reduced macrophage infiltration. To test this, we performed migration assay using conditioned medium (CM) from DMSO- or JQ1-treated mouse CAFs (97f cells). As expected, CM from DMSO treated CAFs (CM-D) induced the migration of RAW264.7 cells, while this effect was reduced using CM from JQ1-treated CAFs (CM-J) (Supplementary Figure S8B).

TAMs can be classified into two subclasses, namely M1 and M2 macrophages. In general, M1 macrophages are characterized by high expression of pro-inflammatory cytokines such as TNF-α, IL-1β, and IL-6. M2 macrophages have been implicated in creating an immunosuppressive microenvironment through immunosuppressive mediators such as arginase-1 (Arg-1), TGF-β, and IL-10 and also through promoting T-cell apoptosis via programmed death ligand 1(PD-L1) expression. To see if the inactivation of CAFs might affect macrophage function and/or polarization, we studied the effects of CAF-CM on bone-marrow-derived macrophages (BMDMs). Interestingly, most genes associated with both M1 and M2 phenotypes were downregulated in BMDMs treated with CM-J (Supplementary Figure S8C). These results highlight the impact of JQ1-mediated inactivation of CAFs on other stromal cells, though the net effect on the tumor microenvironment still requires further investigation.

SUPPLEMENTARY METHODS

Mutation analysis

Genomic DNA was extracted using the DNeasy mini kit (Qiagen). Exon 2 of *KRAS* was amplified by PCR using previously described primers [5]. Mutations in codon 12 of *KRAS* were analyzed by direct sequencing.

siRNA

hCAFs were seeded at 1×10^5 cells per well in a collagen-coated 6-well plate and cultured overnight. Then the cells were transfected with siRNAs at a final concentration of 20 nM using the Lipofectamine RNAi Max Transfection Reagent (Life Technologies). Four days after transfection, cells were harvested and analyzed. The siRNAs used in this study are as follows: siNC (Negative Control siRNA, QIAGEN), siBRD4_1 (s23901, Ambion), and siBRD4_2 (s23902, Ambion).

Cytokine array

Cytokines secreted from CAFs were screened using the RayBio Human Cytokine Antibody Array CYT-2000 kit (RayBiotech Inc.) according to the manufacturer's instructions. CM was prepared as described, and used without concentration.

Chromatin immunoprecipitation (ChIP)-qPCR

ChIP-qPCR was performed as described previously [6] with slight modifications. Briefly, 97f cells were grown in 10 cm plates up to 70-80% confluent. After 24hr of serum starvation in DMEM containing 0.1% FBS, 97f cells were pre-treated with DMSO or 1 µM JQ1 for 2 hours, followed by TGF-β1 (10 ng/mL, 1 to 4 hours). Cells were cross-linked with 1% formaldehyde for 10 minutes at room temperature followed by quenching with 125 mM glycine. After two washes with PBS, cross-linked cells were lysed with lysis buffer 1 (50 mM HEPES-KOH pH 7.5, 140 mM NaCl, 1 mM EDTA, 10% glycerol, 0.5% NP-40, and 0.25% Triton X-100), followed by centrifugation at 1,000 xg for 5 minutes. Pellets were washed with lysis buffer 2 (10 mM Tris-HCl pH 8.0, 200 mM NaCl, 1 mM EDTA pH 8.0, and 0.5 mM EGTA pH8.0). Pellets equivalent to 1×10^7 cells were suspended in 200 µL of sonication buffer (50 mM Tris-HCl pH 8.0, 10mM EDTA pH8.0, 1% SDS) and aliquoted in 1.5 mL TPX microtubes (HTC-M50001, Cosmo Bio). Each aliquot was sonicated

with a sonicator (Bioruptor UCD-250, Cosmo Bio) for 15 cycles at high output (30-second sonication followed by 30-second rest). Lysates were cleared by centrifuging at 20,000xg for 10 minutes at 8°C, and 150 µL cleared lysates (that are equivalent to 7.5 x 10^6 cells) were further diluted to 1.5 mL with ChIP dilution buffer (16.7 mM Tris-HCl pH 8.0, 0.01% SDS, 1.1% Triton X-100, 1.2 mM EDTA pH8.0, and 167mM NaCl) and incubated overnight at 4°C with magnetic beads bound with antibodies. For input DNA, 30 µL cleared lysates (i.e., 20% input) were used. Beads were washed five times with low salt buffer (20 mM Tris-HCl pH 8.0, 0.1% SDS, 1% Triton X-100, 2 mM EDTA pH 8.0, and 150 mM NaCl) and three times with high salt buffer (20 mM Tris-HCl pH 8.0, 0.1% SDS, 1% Triton X-100, 2 mM EDTA pH 8.0, and 500 mM NaCl). DNA was eluted in elution buffer (10 mM Tris-HCl pH 8.0, 300 mM NaCl, 5 mM EDTA pH 8.0, 1% SDS), followed by decrosslinking overnight at 65°C. After RNase treatment and Proteinase K digestion, DNA was extracted using a PCR purification kit (Qiagen). ChIP and input DNA were analyzed on the StepOnePlus Real-Time PCR System (Applied Biosystems) using the FastStart Universal SYBR Green Master (Roche).

Migration assay

The migration assay was performed using Boyden chambers (5 µm pore size, 24 well type, Coaster 3421) according to manufacture's instructions. Briefly, 2 × 10^5 cells in 100 µl of serum free DMEM were seeded in the upper chambers. As a chemoattractant, 500 µl DMEM containing 10% conditioned medium (CM) from DMSO or JQ1-treated mouse CAFs (97f cells) was added to the lower chamber. After 20 h, the migrated cells were stained with Diff-Quick (Sysmex, Kobe, Japan) and counted as cells per 100× field. Three random 100× fields were counted per inserts and averaged.

Bone marrow derived macrophages

Bone marrow derived macrophages (BMDMs) were prepared as described before [7]. Briefly, femurs and tibiae were harvested from legs of C57BL/6 mice and were flushed using a 25-gauge needle. The bone marrow was filtered through a 40 µm strainer and cultured in Non-Treated culture dishes (Iwaki, 1030-150) in high glucose DMEM (Sigma) supplemented with 10% FBS (Invitrogen), 1% penicillin and streptomycin (Invitrogen), and 10 ng/mL mouse Csf-1. After seven days culture to induce macrophage differentiation, BMDMs were seeded into 6-well plates at a density of 20,000 cells/well. After 6 h serum starvation, BMDMs were cultured in serum-free DMEM with 10% concentrated conditioned medium (CM) from mouse CAFs for 24 h. Cells were harvested and subjected to qRT-PCR.

REFERENCES

1. Cui R, Yue W, Lattime EC, Stein MN, Xu Q, Tan XL. Targeting tumor-associated macrophages to combat pancreatic cancer. Oncotarget. 2016; 7:50735-50754. doi: 10.18632/oncotarget.9383.
2. Yoshikawa K, Mitsunaga S, Kinoshita T, Konishi M, Takahashi S, Gotohda N, Kato Y, Aizawa M, Ochiai A. Impact of tumor-associated macrophages on invasive ductal carcinoma of the pancreas head. Cancer Sci. 2012; 103: 2012-20. doi: 10.1111/j.1349-7006.2012.02411.x.
3. Sanford DE, Belt BA, Panni RZ, Mayer A, Deshpande AD, Carpenter D, Mitchem JB, Plambeck-Suess SM, Worley LA, Goetz BD, Wang-Gillam A, Eberlein TJ, Denardo DG, et al. Inflammatory monocyte mobilization decreases patient survival in pancreatic cancer. Clin Cancer Res. 2013; 19: 3404-15. doi: 10.1158/1078-0432.CCR-13-0525.
4. Nywening TM, Wang-Gillam A, Sanford DE, Belt BA, Panni RZ, Cusworth BM, Toriola AT, Nieman RK, Worley LA, Yano M, Fowler KJ, Lockhart AC, Suresh R, et al. Targeting tumour-associated macrophages with CCR2 inhibition in combination with FOLFIRINOX in patients with borderline resectable and locally advanced pancreatic cancer: a single-centre, open-label, dose-finding, non-randomised, phase 1b trial. Lancet Oncol. 2016; 17: 651-62. doi: 10.1016/S1470-2045(16)00078-4.
5. Kondo J, Endo H, Okuyama H, Ishikawa O, Iishi H, Tsujii M, Ohue M, Inoue M. Retaining cell-cell contact enables preparation and culture of spheroids composed of pure primary cancer cells from colorectal cancer. Proc Natl Acad Sci U S A. 2011; 108: 6235-40. doi: 10.1073/pnas.1015938108.
6. Rahl PB, Lin CY, Seila AC, Flynn RA, McCuine S, Burge CB, Sharp PA, Young RA. c-Myc regulates transcriptional pause release. Cell. 2010; 141: 432-45. doi: 10.1016/j.cell.2010.03.030.
7. Shree T, Olson OC, Elie BT, Kester JC, Garfall AL, Simpson K, Bell-McGuinn KM, Zabor EC, Brogi E, Joyce JA. Macrophages and cathepsin proteases blunt chemotherapeutic response in breast cancer. Genes Dev. 2011; 25: 2465-79. doi: 10.1101/gad.180331.111.

SUPPLEMENTARY FIGURES

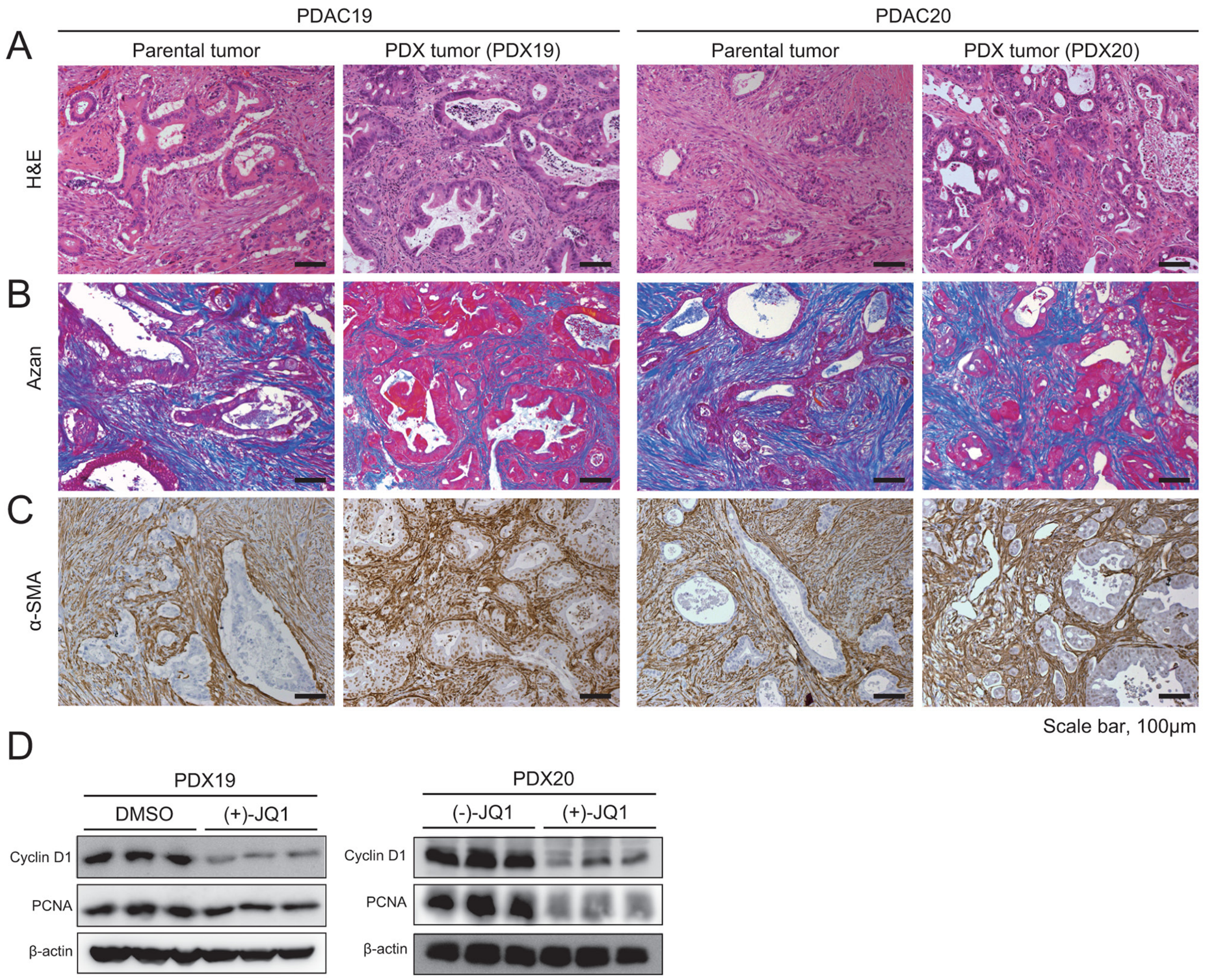

Supplementary Figure S1: Establishment of patient derived tumor xenograft (PDX) from primary PDAC specimens. Histological sections of the original patient sample (parental tumor) and PDX tumors grown subcutaneously in NOD/SCID mice were compared. **A.** H & E staining showed that both parental tumors and subcutaneous xenograft tumors exhibited similar glandular structures that were surrounded by prominent stromal composition, showing a histology of well to moderately differentiated ductal adenocarcinoma. **B.** Azan staining revealed a similar desmoplastic reaction in parental and PDX tumors. **C.** Immunohistochemistry for the CAF activation marker α-SMA. Both primary and PDX tumors show abundant infiltration of activated CAFs into the stroma. Scale bars represent 100 μm. **D.** Related to Figure 1, western blot analysis was performed using bulk tumor lysates for the expression of the proliferation markers, cyclin D1 and PCNA. PCNA and cyclin D1 expression was decreased in JQ1-treated tumors. β-actin was used as a loading control. Three tumors per group were analyzed.

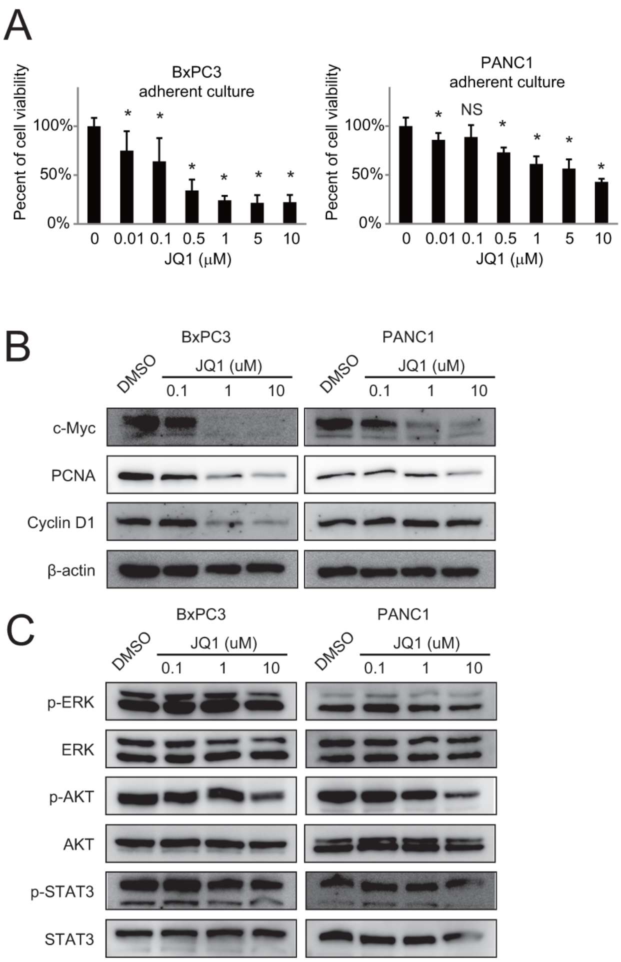

Supplementary Figure S2: Related to Figure 2, the effects of JQ1 on established pancreatic cancer cell lines were evaluated. A. BxPC3 and PANC1 cells were incubated at indicated doses of JQ1 for 72 h, and then cell growth was quantified. *, $P < .05$ compared with vehicle by Student's *t*-test. **B** and **C.** Western blot of whole cell lysates from BxPC3 and PANC1 cells that were treated with JQ1 for 24 h *in vitro*.

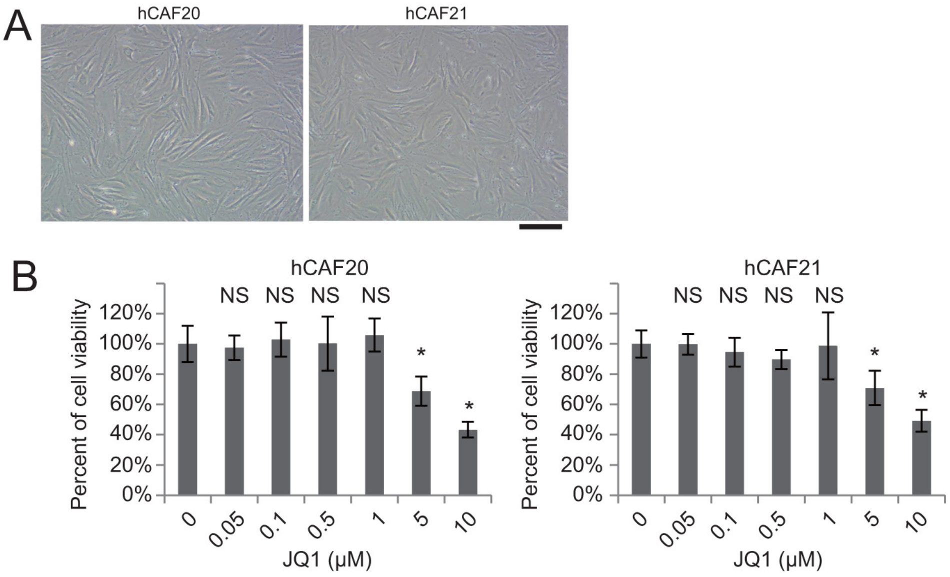

Supplementary Figure S3: A. Representative images of primary human CAFs (hCAF20 and hCAF21) isolated from two PDAC patients who underwent surgical resection. Scale bar represents 100 µm. **B.** hCAFs were treated with the indicated doses of JQ1 for 72 h and cell viability was measured. *, $P < .05$ compared with vehicle (DMSO) by Student's *t*-test. Note that JQ1 did not affect the cell viability of CAFs at doses under 1 µM.

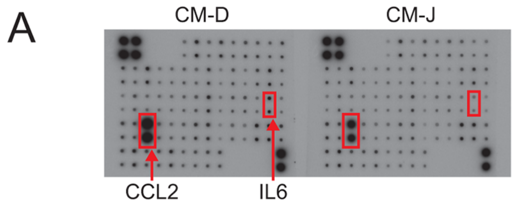

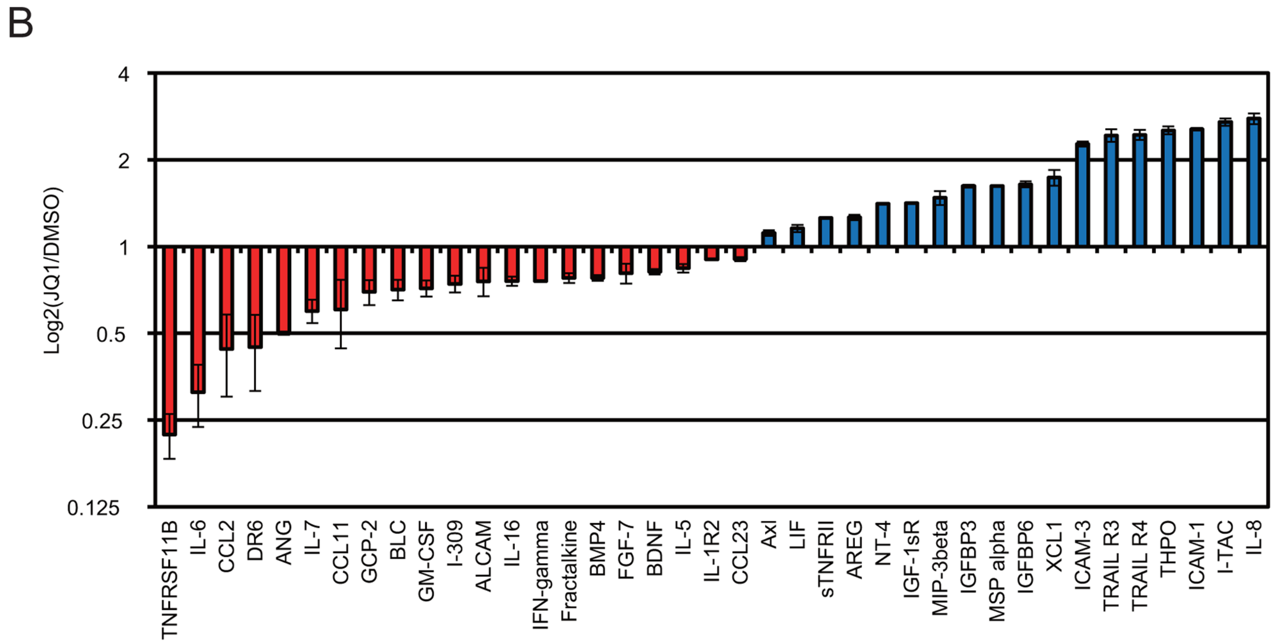

Supplementary Figure S4: Cytokine arrays showing cytokine secretion in CM from DMSO- or JQ1-treated hCAF20 cells (CM-D and CM-J, respectively). A. One of three membranes is shown. **B.** Bars represent the fold change of intensity (log2) on CM-J versus CM-D. Cytokines showing decreased (red) or increased (blue) expression, with statistical significance of p values less than 0.05, are shown.

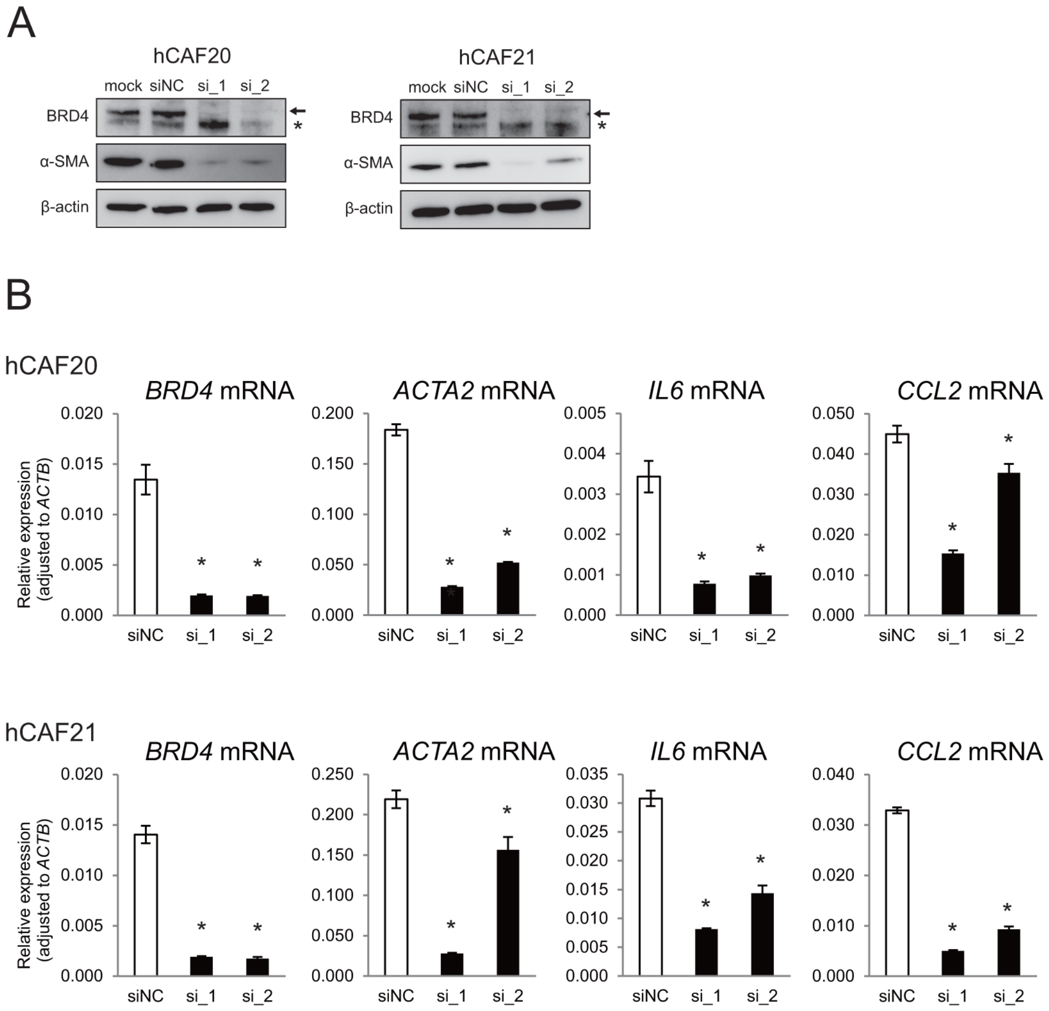

Supplementary Figure S5: siRNA-mediated BRD4 knockdown recapitulated the effect of JQ1 on CAFs. A. Western blotting was performed on hCAFs 96 h after siRNA-mediated knockdown of BRD4. In hCAF 20 and hCAF21 cells, BRD4 expression is sufficiently reduced by both si_1 and si_2 siRNAs, compared with mock transfection (mock) or negative control siRNA (siNC) treated CAFs. Notably, BRD4 knockdown also reduced α-SMA expression in hCAFs. Arrows indicate BRD4. Asterisks indicate non-specific bands. **B.** Quantitative RT-PCR analysis showed that BRD4 knockdown reduced mRNA expression of genes related to activated CAFs. Bars represent means ± SEM (n = 3); *, $P < .05$, compared with siNC.

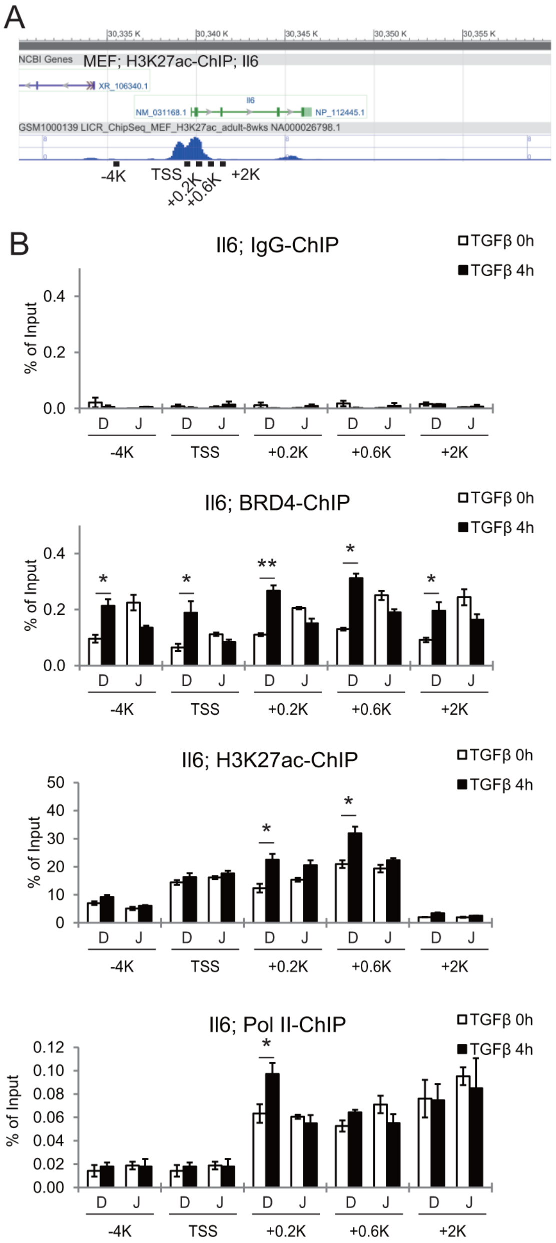

Supplementary Figure S6: Related to Figure 6, ChIP data on Il6 gene was shown. A. Tracks are H3K27ac ChIP-seq data from MEF (ENCODE). Schematic representations of the mouse Il6 gene and ChIP primers were shown. Localization of primers is depicted as distances from the TSS. **B.** After pretreatment with DMSO (D) or JQ1 (J) for 2 h, 97f cells were stimulated with TGF-β1 (10ng/mL) for 0 or 4 h and subjected to ChIP-qPCR. Bars represent means ± SEM (n = 3); *, $P < .05$; **, $P < .01$.

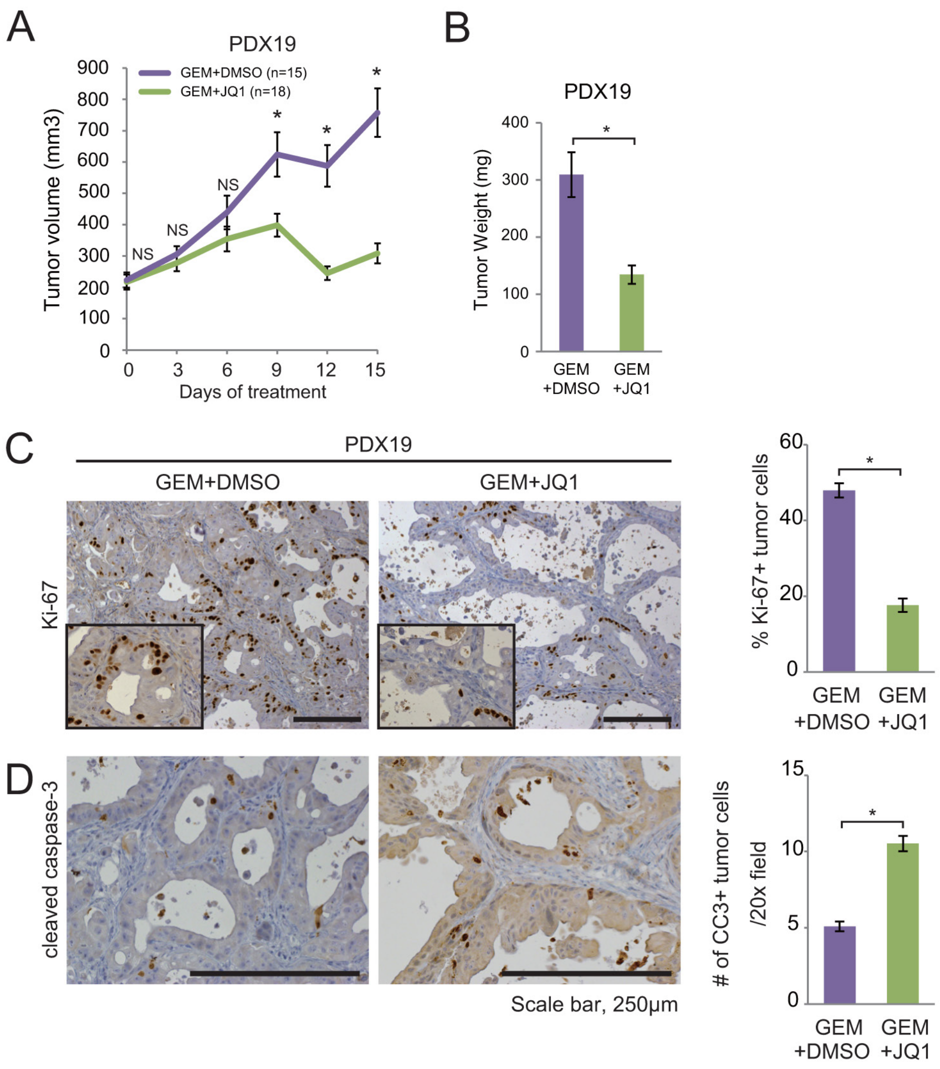

Supplementary Figure S7: The combination of JQ1 and gemcitabine showed additional efficacy over gemcitabine monotherapy. Mice bearing PDX tumors (PDX19) were treated with either gemcitabine (75mg/kg, twice a week, i.p.) plus DMSO or gemcitabine plus JQ1 (50mg/kg, every day, i.p. for 2 wks), **A.** Average volumes of subcutaneous PDX tumors. *, $P < .05$; NS, not significant. **B.** Tumor weight at the end of the treatment period. Bars represent means ± SEM; *, $P < .05$. **C** and **D.** Representative IHC images stained for Ki-67 (C) and cleaved caspase-3 (CC3) (D). **(C)** Proliferation of tumor cells was quantified as the percentage of Ki-67 positive tumor cells per 20x field (average of 5 random fields per tumor). Eight tumors per group were analyzed. *, $P = 1.4 \times 10^{-8}$. **(D)** Apoptosis of tumor cells was quantified as the number of CC3 positive tumor cells per 20x field (average of 5 random fields per tumor). Eight tumors per group were analyzed. *, $P = 3.1 \times 10^{-5}$.

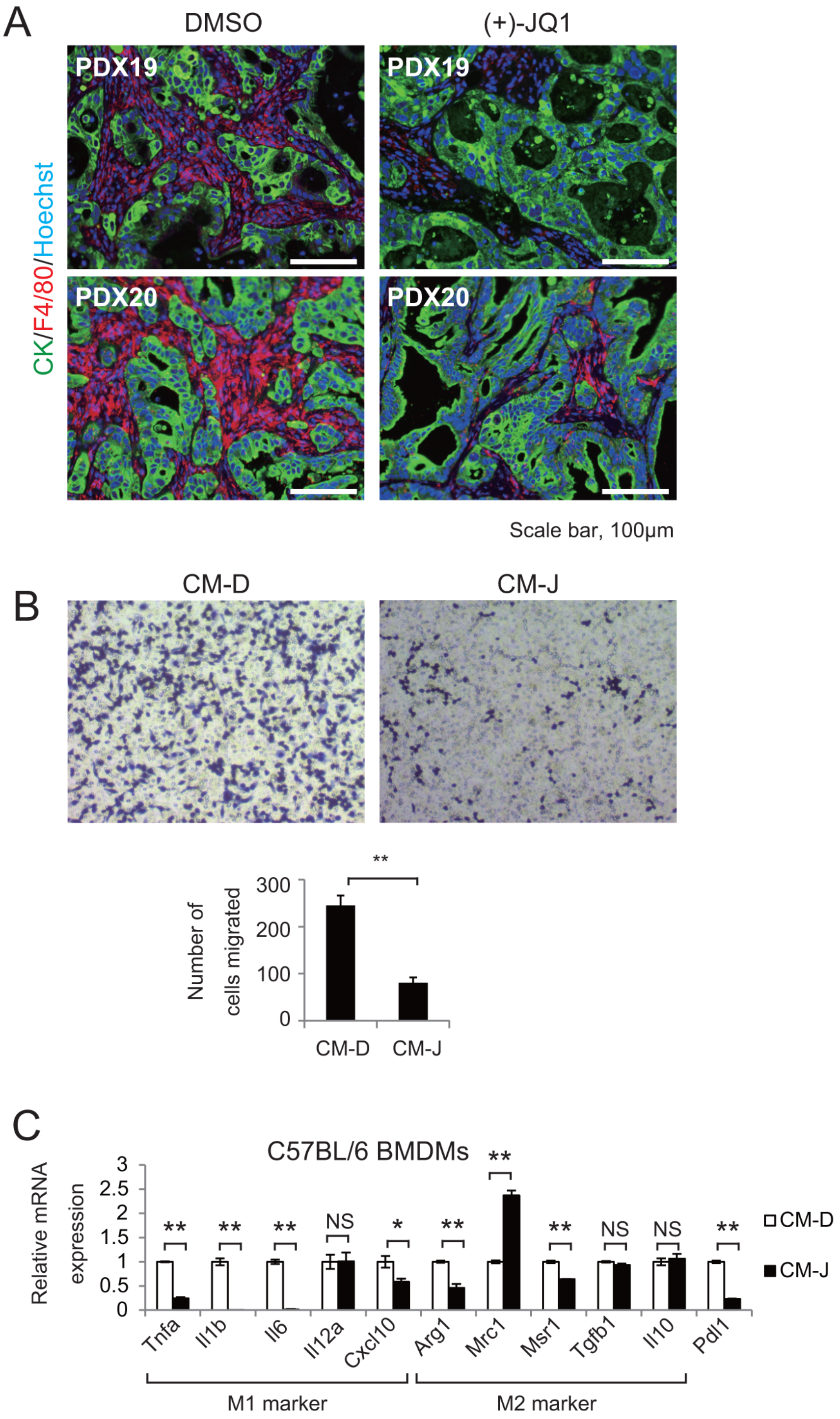

Supplementary Figure S8: JQ1 impairs the ability of CAFs to recruit macrophages and to modulate their function. A. Representative immunofluorescence images of PDX tumors that were treated with vehicle or JQ1 for 2 wks. Fluorescent images showing staining with pan-cytokeratin (CK, green) and F4/80 (red). Nuclei were stained with Hoechst (blue). Scale bars represent 100 μm. **B.** Migration assay of RAW264.7 cells. RAW264.7 cells were seeded onto the upper chambers. Conditioned medium (CM) from DMSO-treated and JQ1-treated 97f mouse CAFs (CM-D and CM-J, respectively) were used as chemoattractant. The scale bar represents 100 μm. Bars represent means ± SEM (n = 3); **, *P* < .01, compared by Student's t-test. **C.** Quantitative RT-PCR of selected genes in BMDMs that were cultured with CM for 24 h.

SUPPLEMENTARY TABLES

Supplementary Table S1: Clinicopathological characteristics of primary pancreatic cancer specimens

Patient ID	Diagnosis	Surgical procedures	Age	Sex	Pathology	KRAS status in codon 12	Establishment of PDX	Establishment of primary CAF
**PDAC19**	PDAC	DP	44	Male	Well/Mod	GAT(mt)/GGT(wt)	Yes (PDX19)	No
**PDAC20**	PDAC	PD	78	Male	Well/Mod	GAT(mt)/GGT(wt)	Yes (PDX20)	Yes (hCAF20)
**PDAC21**	PDAC	PD	53	Male	Mod	not analyzed	No	Yes (hCAF21)

Supplementary Table S2: Lists of antibodies.

See Supplementary File 1

Supplementary Table S3: Lists of primers for qRT-PCR.

See Supplementary File 2

Supplementary Table S4: Lists of primers for ChIP-qPCR

Target	Forward primer (5' to 3')	Reverse primer (5' to 3')
**Gli1_TSS1**	TTTTTCTCGCTGTTGCCACC	GAACGGTCCGAAGGAAGGAT
**Gli1_TSS2**	TATGGGGTTGGGAGAGTTTG	AAAGAGACCTGGGACAGACAC
**Col1a1_-10K**	GCTGGGTTGGCTCCAGTAAA	GGGGTGCAAGCAACCTTTTC
**Col1a1_-2K**	TTGGGGATGCTAGATTGGGAG	CAGGGTAGAATGCCACGAAG
**Col1a1_TSS**	GGAGTTTCTCCTCGGGACGG	TACTGTCTTCTTGGCCATGCG
**Col1a1_+0.5K**	GCTGCGGGATGATTCATAAGG	CGTAGTGCTCTCGGCTGG
**Col1a1_+6K**	TCCAGGTCCCAAGGGTAACA	AGGTTCACCCTGTGTAGGGA
**Il6_-4K**	TTGCTATCCATAGAGCCACTCCTA	GCCTTACAGCCTCAGTGTTGC
**Il6_TSS**	ACCGCTATGAAGTTCCTCTCTG	CAGTCTCAATAGCTCCGCCAG
**Il6_+0.2K**	GCCTTCTTGGGACTGATGCT	GACAGGTCTGTTGGGAGTGG
**Il6_+0.6K**	GTTTTGAGTGGAGGTTGGGAAG	ACAGAGAATGGCCCACTGTGAAT
