# Supplementary material for: Stromal remodeling by the BET bromodomain inhibitor JQ1 suppresses the progression of human pancreatic cancer
Source: Oncotarget. 2016 Aug 9;7(38):61469–84. doi: 10.18632/oncotarget.11129 (PMC5308665; doi:10.18632/oncotarget.11129)
Supplement: Supplementary file 2 [file oncotarget-07-61469-s002.docx]

**Supplementary Table S2**. Lists of antibodies.

**IHC**

| Antibody | Manufacturer | Catalog No. | Origin | Dilution | Antigen retrieval |
| --- | --- | --- | --- | --- | --- |
| FSP1 | abcam | ab27957 | Rabbit | 1:200 | Citrate buffer, boil |
| αSMA | DAKO | M0851 | Mouse | 1:100 | Citrate buffer, boil |
| Ki-67 | DAKO | M7240 | Mouse | 1:100 | Citrate buffer, boil |
| phospho-STAT3 | Cell Signaling Technology | CST9145 | Rabbit | 1:100 | 10mM EDTA pH 8.0, autoclave |
| phospho-ERK | Cell Signaling Technology | CST4376 | Rabbit | 1:100 | Citrate buffer, autoclave |
| cleaved Caspase-3 | Cell Signaling Technology | CST9661 | Rabbit | 1:100 | Citrate buffer, autoclave |

**IF**

| Antibody | Manufacturer | Catalog No. | Origin | Dilution |
| --- | --- | --- | --- | --- |
| αSMA | DAKO | M0851 | Mouse | 1:100 |
| FSP1 | Abcam | ab27957 | Rabbit | 1:100 |
| FN | BD Transduction Laboratories | BD610077 | Mouse | 1:100 |
| pan-Cytokeratin | Santa Cruz | sc-15367 | Rabbit | 1:50 |
| F4/80 | AbD Serotec | MCA497GA | Rat | 1:100 |

**WB**

| Antibody | Manufacturer | Catalog No. | Origin | Dilution | Dilution (2nd Abs) | Blocking reagents. |
| --- | --- | --- | --- | --- | --- | --- |
| phospho-STAT3 | Cell Signaling Technology | CST9145 | Rabbit | 1:1000 | 2nd 1:5000 | 5%BSA/TBST |
| STAT3 | Cell Signaling Technology | CST9132 | Rabbit | 1:2000 | 2nd 1:5000 | 5%BSA/TBST |
| phospho-ERK | Cell Signaling Technology | CST9101 | Rabbit | 1:1000 | 2nd 1:5000 | 5%BSA/TBST |
| ERK | Cell Signaling Technology | CST9102 | Rabbit | 1:2000 | 2nd 1:5000 | 5%BSA/TBST |
| phospho-Akt | Cell Signaling Technology | CST9271 | Rabbit | 1:1000 | 2nd 1:5000 | 5%BSA/TBST |
| Akt | Cell Signaling Technology | CST9272 | Rabbit | 1:2000 | 2nd 1:5000 | 5%BSA/TBST |
| c-Myc | Santa Cruz | sc-40 | Mouse | 1:1000 | 2nd 1:5000 | 5% skim milk/TBST |
| PCNA | Santa Cruz | sc-7907 | Rabbit | 1:1000 | 2nd 1:5000 | 5% skim milk/TBST |
| Cyclin D1 | Santa Cruz | sc-2044 | Mouse | 1:1000 | 2nd 1:5000 | 5% skim milk/TBST |
| cleaved Caspase-3 | Cell Signaling Technology | CST9661 | Rabbit | 1:1000 | 2nd 1:5000 | 5% skim milk/TBST |
| α-SMA | DAKO | M0851 | Mouse | 1:5000 | 2nd 1:10000 | 5% skim milk/TBST |
| β-actin | Sigma | A5441 | Mouse | 1:10000 | 2nd 1:10000 | 5% skim milk/TBST |
| BRD4 | Bethyl Laboratories | A301-985A100 | Rabbit | 1:10000 | 2nd 1:5000 | 5% skim milk/TBST |
| GLI1 | Cell Signaling Technology | CST2553 | Rabbit | 1:1000 | 2nd 1:5000 | 5% skim milk/TBST |
| phospho-SMAD3 | Cell Signaling Technology | CST9520 | Rabbit | 1:1000 | 2nd 1:10000 | 5%BSA/TBST |
| SMAD3 | Cell Signaling Technology | CST9523 | Rabbit | 1:2000 | 2nd 1:10000 | 5%BSA/TBST |
| Lamin B1 | Santa Cruz | sc-20682 | Rabbit | 1:1000 | 2nd 1:10000 | 5% skim milk/TBST |
