# Supplementary material for: Stromal remodeling by the BET bromodomain inhibitor JQ1 suppresses the progression of human pancreatic cancer
Source: Oncotarget. 2016 Aug 9;7(38):61469–84. doi: 10.18632/oncotarget.11129 (PMC5308665; doi:10.18632/oncotarget.11129)
Supplement: Supplementary file 3 [file oncotarget-07-61469-s003.docx]

**Supplementary Table S3**. Lists of primers for qRT-PCR.

| **Target gene** | **Forward primer (5’ to 3’)** | **Reverse primer (5’ to 3’)** |
| --- | --- | --- |
| **human ACTB** | CTGGAACGGTGAAGGTGACA | AAGGGACTTCCTGTAACAACGCA |
| **human COL1A1** | CTGGCCTCCCTGGAATGAAG | TGGGGACCTTCAGAGCCT |
| **human COL1A2** | CAGCAGGAGGTTTCGGCTAA | GCTGGGCCCTTTCTTACAGT |
| **human COL3A1** | TGCCCTACTGGTCCTCAGAA | TCGTCCGGGTCTACCTGATT |
| **human FN1** | CAGTGGGAGACCTCGAGAAG | TCCCTCGGAACATCAGAAAC |
| **human ACTA2** | CCGGGAGAAAATGACTCAAA | GCGTCCAGAGGCATAGAGAG |
| **human IL6** | CACACAGACAGCCACTCACC | TTTTCTGCCAGTGCCTCTTT |
| **human CCL2** | AGCCACCTTCATTCCCCAAG | CTCCTTGGCCACAATGGTCT |
| **human FGF2** | GGTGAAACCCCGTCTCTACA | ACCTTGACCTCTCAGCCTCA |
| **human FGF7** | GAAAGGCTCAAGTTGCACCAG | TGCTGTGACGCTGTTTGCTA |
| **human FGF9** | AGCCGATTTGGCATTCTGGA | TCCACGTGCTTATATAGGTTTGA |
| **human EGF** | TGCAGAGGGATACGCCCTAA | TGCGTGGACAGGAAACAAGT |
| **human PDGFA** | GGCCAAGGTGGAATACGTCA | CTCACATCTGGTTGGCTGCT |
| **human BRD4** | AGGCAAAAGGAAGAGGACG | CGATGCTTGAGTTGTGTTTGG |
| **human GLI1** | AAGGAATTCGTGTGCCATTGGG | ACATGTAAGGCTTCTCACCCGT |
| **mouse Actb** | CTGTCGAGTCGCGTCCA | ACCCATTCCCACCATCACAC |
| **mouse Acta2** | GACATCAGGAAGGATCTCTATGC | GCTGATCCACAAAACGTTCACA |
| **mouse Il6** | CACTTCACAAGTCGGAGGCT | CTGCAAGTGCATCATCGTTGT |
| **mouse Ccl2** | AGTTAACGCCCCACTCACCT | GACCCATTCCTTCTTGGGGTC |
| **mouse Fgf2** | GCCAACCGGTACCTTGCTAT | ACTGCCCAGTTCGTTTCAGT |
| **mouse Fgf7** | GCAGACACGGAACTCTTGTG | CCCACTAGACAGACGAGGTG |
| **mouse Fgf9** | TCGCCTAGTGTCTCCTGGTTGATA | ACCAGGCCCACTGCTATACTG |
| **mouse Egf** | AAAGAACTCTCCCGGAGCC | CCATCTGGGTCAATCCGAGAG |
| **mouse Pdgfa** | TGTTGTAACACCAGCAGCGT | ACCTCACATCTGTCTCCTCCTCC |
| **mouse Gli1** | AAGGAATTCGTGTGCCATTGGG | ACATGTAAGGCTTCTCACCCGT |
| **mouse Tnfa** | ATCGGTCCCCAAAGGGATGA | GCTACAGGCTTGTCACTCG |
| **mouse Il1b** | TGCCACCTTTTGACAGTGATG | ATGTGCTGCTGCGAGATTTG |
| **mouse Il12a** | GCTGAAATCTTCTCACCGTGC | GTGGTTTAGGAGGGCAAGGG |
| **mouse Cxcl10** | CCTATCCTGCCCACGTGTTG | TCTCTGCTGTCCATCCATCG |
| **mouse Arg1** | CTTGCGAGACGTAGACCCTG | CTTCCTTCCCAGCAGGTAGC |
| **mouse Mrc1** | ATGGATTGCCCTGAACAGCA | TGTACCGCACCCTCCATCTA |
| **mouse Msr1** | AGAGAAGGGGAGTGTAGGCG | GATGATAGTAGGGTGCTCTGCC |
| **mouse Tgfb1** | CTGCTGACCCCCACTGATAC | GTGAGCGCTGAATCGAAAGC |
| **Mouse Il10** | GCTATGCTGCCTGCTCTTAC | TGTCCAGCTGGTCCTTTGTTT |
| **Mouse Pdl1** | CACAGCCTGCTGTCACTTGC | AGGGAATCTGCACTCCATCG |
